# Sustainable wood electronics by iron-catalyzed laser-induced graphitization for large-scale applications

**DOI:** 10.1038/s41467-022-31283-7

**Published:** 2022-06-27

**Authors:** Christopher H. Dreimol, Huizhang Guo, Maximilian Ritter, Tobias Keplinger, Yong Ding, Roman Günther, Erik Poloni, Ingo Burgert, Guido Panzarasa

**Affiliations:** 1 Wood Materials Science, Institute for Building Materials, ETH Zürich, 8093 Zürich, Switzerland; 2 Cellulose & Wood Materials Laboratory, Empa, 8600 Dübendorf, Switzerland; 3 Laboratory of Adhesives and Polymer Materials, Institute of Materials and Process Engineering, ZHAW Zürich University of Applied Sciences, 8401 Winterthur, Switzerland; 4 Multifunctional Materials, Department of Materials, ETH Zürich, 8093 Zürich, Switzerland; 5 Complex Materials, Department of Materials, ETH Zürich, 8093 Zürich, Switzerland; 6 Present Address: High Enthalpy Flow Diagnostics Group, Institute of Space Systems, University of Stuttgart, 70569 Stuttgart, Germany

**Keywords:** Electronic devices, Electronic properties and devices, Design, synthesis and processing

## Abstract

Ecologically friendly wood electronics will help alleviating the shortcomings of state-of-art cellulose-based “green electronics”. Here we introduce iron-catalyzed laser-induced graphitization (IC-LIG) as an innovative approach for engraving large-scale electrically conductive structures on wood with very high quality and efficiency, overcoming the limitations of conventional LIG including high ablation, thermal damages, need for multiple lasing steps, use of fire retardants and inert atmospheres. An aqueous bio-based coating, inspired by historical iron-gall ink, protects wood from laser ablation and thermal damage while promoting efficient graphitization and smoothening substrate irregularities. Large-scale (100 cm^2^), highly conductive (≥2500 S m^−1^) and homogeneous surface areas are engraved single-step in ambient atmosphere with a conventional CO_2_ laser, even on very thin (∼450 µm) wood veneers. We demonstrate the validity of our approach by turning wood into highly durable strain sensors, flexible electrodes, capacitive touch panels and an electroluminescent LIG-based device.

## Abstract (web summary)

Ecologically friendly wood electronics will help alleviating the shortcomings of state-of-art cellulose-based green electronics. Here, the authors introduce iron-catalyzed laser-induced graphitization (IC-LIG) as an innovative approach for engraving large-scale electrically conductive structures on wood with high quality and efficiency.

## Introduction

Developing electronic devices from renewable and biodegradable materials using environmentally friendly manufacturing routes (“green electronics”) is mandatory to meet the demands of a sustainable society^1^. The foreseen implementation of the Internet-of-Things (IoT) approach to smart buildings and even cities poses unmet challenges in terms of scale and durability of sustainable electronic materials^2,3^. State-of-art green electronics is nowadays dominated by relatively small, disposable devices made from (nano-)cellulose-based materials^4–6^. However, their sustainability may be challenged by the many demanding steps, in terms of amount of energy and chemicals, needed for the isolation and reassembly of cellulose into functional materials. Using wood as substrate for electronic devices can help solving this problem at the root. Wood materials are also especially useful for applications requiring not only high mechanical strength and scalability, such as structural health monitoring (e.g., strain sensors incorporated in load-bearing structures), but also valuable esthetics and haptics (such as touch-screens and light displays as human-machine interfaces in smart buildings).

Wood is a renewable and biodegradable CO_2_-storing natural resource, an excellent state-of-art building material with highly appreciated esthetics and haptics, lightweight but with high mechanical strength. The development of wood electronics has so far been limited by the complex wood structure and the lack of intrinsic electrical conductivity. Previous attempts toward electrically conductive wood materials have included the surface coating with metal nanowires^7^ and carbon-based inks^8^, as well as bulk impregnation e.g. with low-melting metals^9^. In these approaches, irrespective of their limited sustainability, wood has been used as a passive substrate. As for other biological substrates, graphitizing wood under proper conditions can result in graphene- and graphite-like materials with reasonable electrical properties (>500 S m^−1^ and <1 kΩ ﻿◻^−1^)^10–13^. However, this usually happens at the expense of structural and mechanical integrity. Finding a way to confine graphitization selectively at the wood surface, down to several microns but leaving the bulk intact, would open up new avenues for wood electronics.

Laser-induced graphitization (LIG) has been used to convert a variety of inorganic^14,15^ and organic precursors into electrically conductive materials^16–18^. This graphitization process can be best described as a combined photothermal and photochemical conversion of a precursor that leads to a porous carbonaceous material. LIG is a cost-effective technique featuring high processing speeds and flexibility, making possible to combine laser-engraving of graphitized patterns with controlled morphology^19^ together with laser-cutting. First attempts of biological materials laser-induced graphitization^16,20^ led to products with reasonable, yet not entirely sufficient, electric and structural properties for most envisaged applications, such as large-scale sensors and actuators.

Wood is a challenging material for laser-induced graphitization. Due to the low thermal conductivity of wood (~0.2 W m^−1^ K^−1^)^21^, its surface can undergo significant thermal degradation well before the bulk could reach the decomposition temperature, resulting in asymmetric shrinkage and mechanical stresses that result into cracks^10^. This problem is frequently encountered when highly localized heat sources are applied, as in laser-induced graphitization. Nevertheless, high temperatures (1200–3000 °C) are required to convert wood into graphite-like materials with reasonable electrical properties^13,20^. To reduce thermal damage and ablation rates, the lasing can be performed under an oxygen-free atmosphere (Ar or H_2_)^16^. Alternatively, the wood surface can be graphitized to a dense char layer as a barrier against heat and mass transport, which is subsequently made electrically conductive via LIG^17^. So far, this two-step approach for LIG in ambient atmosphere has only been reported for wood and cellulose-based materials (paper and fabrics) impregnated with a fire-retardant (boric acid). Although slow engraving speeds, reduced power values, and multiple (up to five) lasing steps were employed, the resulting LIG structures were still inhomogeneous and showed numerous cracks^17^. Photo-assisted graphitization of native wood by means of femtosecond laser systems was suggested to minimize thermal damage, but satisfactory electrical conductivity could only be achieved with low engraving speeds (from 5 to 15 mm s^−1^) resulting in disproportionate process times. Even under these conditions, substrate ablation could only be reduced to around 300–500 µm^20^, causing excessive damage to thin (500–1500 µm) decorative wood veneers.

**Fig. 1:**
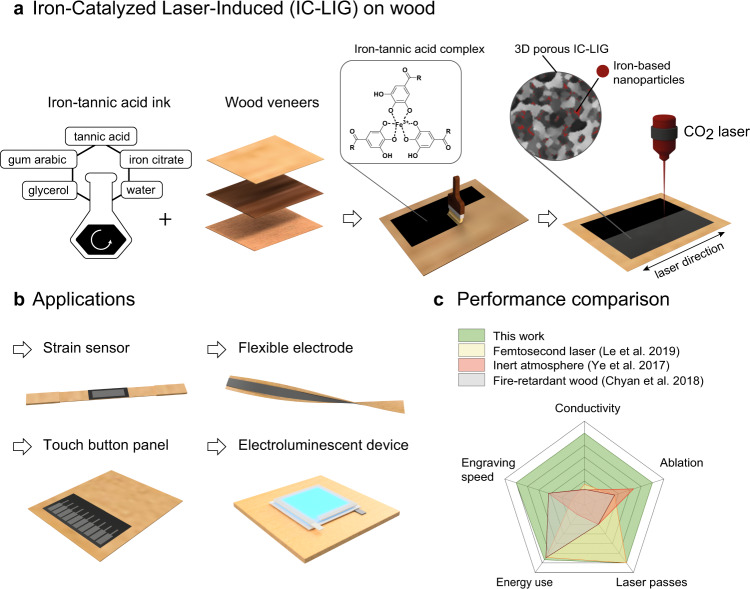
Iron-catalyzed laser-induced graphitization (IC-LIG) of wood and applications thereof. **a** Schematic representation of the IC-LIG process. The substrate (e.g., a wood veneer) is coated with an iron-tannic acid ink (the inset shows a simplified structure of the iron-tannic acid complex contained in the ink, see also Supplementary Fig. 1). The ink-coated wood veneer is then treated with a CO_2_ laser to achieve IC-LIG (the inset shows a schematic of the resulting 3D porous iron-carbon composite). **b** Proof-of-concept applications demonstrated in this work for IC-LIG structures on wood veneers: a strain sensor, a flexible electrode, a touch buttons panel, and an electroluminescent device. **c** A radar plot comparing the performance of our IC-LIG with other previously published LIG approaches in terms of process parameters (faster engraving speed, lower energy use, only one laser pass needed) and quality (higher electrical conductivity, lower substrate ablation) of the resulting LIG materials (see also Supplementary Table 1).

Here we demonstrate an innovative and convenient method for engraving highly conductive (≥20 Ω ﻿◻^−1^ and up to 2500 S m^−1^) LIG patterns on the surface of thin wood veneers, with a single lasing step under ambient atmosphere, using a conventional CO_2_ laser source and high writing speeds. This approach, iron-catalyzed laser-induced graphitization (IC-LIG), takes advantage of the intumescent and thermo-catalytic properties of an iron-tannic acid ink of our formulation (Fig. 1a). Thanks to our approach, electrically conductive graphite-like structures can be engraved even on thin wood veneers (~0.4–1.5 mm) and paper substrates without ablation and thermal damage. This allows the fabrication of a variety of devices directly on wood, including the first reported example of an electroluminescent device made with a LIG electrode (Fig. 1b). Compared to recent LIG reports, we achieved conductivity values of up to 2500 S m^−1^ on different wood substrates, an order of magnitude higher than the highest literature value (400 S m^−1^)^20^, with increased engraving speed (up to 35 times faster) and reduced energy consumption thanks to lasing only a single time with moderate laser power (Fig. 1c, Supplementary Table 1). These features make IC-LIG a highly efficient laser-induced graphitization method. We analyzed in detail the relevant electrical, morphological and compositional characteristics of the resulting LIG structures using state-of-art techniques, including 4-point probe measurements, Raman spectroscopy, optical and electron microscopy, and wide-angle X-ray diffraction. Furthermore, we demonstrated for the first time the homogeneity of the electrical properties of the obtained LIG-wood, mapping it over a very large area (100 cm^2^) using an innovative eddy-current measurement technique. To showcase the usefulness of IC-LIG for sustainable large-scale wood electronics, we developed four proof-of-concept applications, namely: a highly durable strain sensor suitable for structural health monitoring, a flexible electrode for motion tracking, a human-machine interface (capacitive touch panel) with the esthetics and haptics of wood, and the first example of an electroluminescent device made using LIG as electrode material.

## Results

Complexes of iron cations and polyphenols (such as tannic acid) have received increasing attention over the last decade thanks to their sustainability, biocompatibility and rich chemistry, making them of interest for a variety of applications ranging from functional coatings to the assembly of nanostructures^22,23^. Many of these researches, including the present one, have been inspired by the iron-gall ink used in Europe for writing manuscripts since the Middle Ages^24,25^. We chose tannic acid (TA) as a readily available natural polyphenol with well-known iron-complexing ability^26^ and intumescent thermal behavior^27^. Mixing iron(III) citrate with excess tannic acid results in the instantaneous formation of insoluble complexes with a characteristic deep bluish-purple color (Supplementary Fig. 1)^28^. Compared to more common chloride, sulfate or nitrate salts, the use of iron(III) citrate helps to avoid the generation of hazardous gases during lasing and the uncontrolled introduction of heteroatomic dopants. Further addition of gum arabic facilitates to stabilize the complex in suspension^29^, resulting in a stable ink, while glycerol reduces crack formation upon drying.

The visual appearance of different native and ink-coated wood and paper substrates before and after a single laser passage is compared in Supplementary Fig. 2. All the uncoated substrates were severely damaged (some were completely incinerated, like balsa wood and the cellulose paper we used as control), while for the ink-coated ones the laser treatment resulted in a homogeneous carbonaceous layer with no visible cracks. The ink was deposited on all samples with a paintbrush. On wood veneers, it was found to penetrate on average only into the first cell layers (≤50 µm), forming a layer of variable thickness (between 20 and 80 µm depending on the wood species and surface roughness, Supplementary Fig. 3a, b) which smoothened the otherwise naturally irregular wood surface. By contrast, paper was almost completely impregnated.

**Fig. 2:**
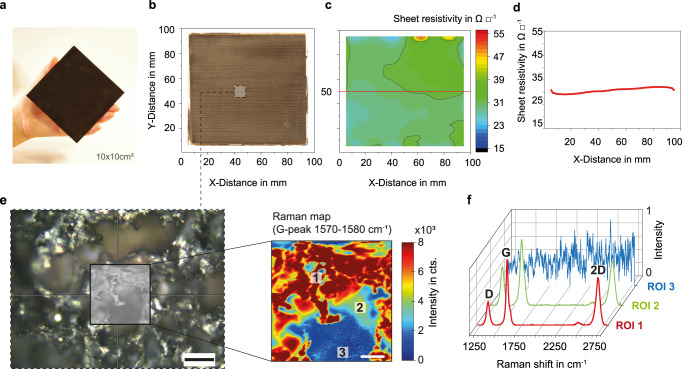
Characterization of IC-LIG structures on wood: electrical conductivity and Raman spectroscopy. **a**, **b** Optical images of a large-scale (100 cm^2^) sample of IC-LIG on spruce wood, and **c** its sheet resistivity map (measured with the contactless eddy-current method; see also Supplementary Fig. 4). **d** The horizontal line plot at *y* = 50 mm highlights that the sheet resistivity is constant over a 10 cm distance. **e** Optical image of the graphitized porous structure and associated Raman map (*λ* =  532 nm, scale bar 50 µm) of the G-peak (1570–1580 cm^−1^). **f** Regions of interest (ROI, −15 × 15 µm^2^ each) measured at three different positions.

We laser-treated large (~100 cm^2^) samples (Fig. 2a, b), then measured their sheet resistivity values using both a conventional four-point probe setup (Supplementary Fig. 3c) and a contactless, non-destructive eddy-current method (Fig. 2b and Supplementary Figs. 4 and 5). The values obtained with both techniques were in excellent agreement and confirmed the successful production of highly conductive materials for every tested wood species as well as for paper (Supplementary Fig. 3c). Despite the intrinsic structural anisotropy of wood substrates, no significant differences in sheet resistivity could be detected by performing the measurements in the direction parallel or perpendicular to the lasing direction as well as to the wood fiber direction (Supplementary Fig. 3c), suggesting that neither the wood substrate nor the lasing direction could negatively affect the outcome of our process. The high uniformity of laser-treated areas was further demonstrated by the two-dimensional sheet resistivity maps obtained with eddy-current measurements (Fig. 2c, d and Supplementary Fig. 4). We point out that this is the first time that the homogeneity of such a large-area (100 cm^2^) LIG surface is showed by a direct measurement.

A thorough analysis of characteristic Raman peaks^16,30–32^ confirmed the presence of graphite-like materials in the laser-treated areas. Graphite-like carbon generally shows three distinct peaks in its Raman spectrum, the D-peak (~1350 cm^−1^, associated with the breathing mode of *sp*^2^ atoms), the G-peak (~1580 cm^−1^, due to the bond stretching of *sp*^2^ atoms pairs), and the 2D-peak (~2970 cm^−1^, an overtone of the D band)^30,33,34^. To prove the successful graphitization of ink-coated samples, we mapped the G-peak band (1570–1580 cm^−1^, Fig. 2e) since the G-peak is always observed for graphitic materials (*sp*^2^ carbon systems). The dark red areas in the map are due to high-intensity G-peak bands, as can be seen from the corresponding region of interest (ROI, ~15 × 15 µm^2^) in Fig. 2f (ROI-1). The blue areas, which do not show any Raman signal associated to graphene- or graphite-like materials (Fig. 2f, ROI-3), are empty holes in a porous structure as revealed by both optical and scanning electron microscopy (SEM).

**Fig. 3:**
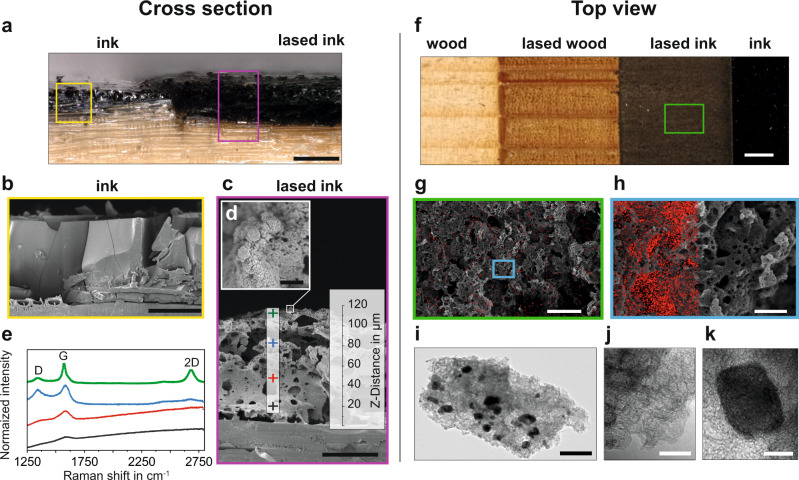
Characterization of IC-LIG structures on wood: morphological and structural analysis. **a** Image showing lased and pristine ink-coated spruce veneer from the cross section perspective. SEM images of (**b**) pristine and (**c**) lased ink-coated spruce. The inset **d** is a magnified image of the upper surface showing its nanostructure. In **c**, crosses of different colors indicate the typical Raman measurement points on samples treated with high fluence parameters. The corresponding Raman spectra are shown in **e**. The top view optical image in **f** compares uncoated (left) with ink-coated (right) spruce after laser treatment. **g**, **h** SEM images (top view) showing the multi-hierarchical porosity of lased ink-coated spruce. **i**–**k** TEM images of the iron-carbon composite. The dark spots are due to the iron-rich phase. Scale bars: **a** 200 µm, **b** and **c** 50 µm, **d** 1 µm, **f** 10 mm, **g** 100 µm, **h** 25 µm, **i** 20 nm, **j**, **k** 10 nm.

As shown in Fig. 3a–d, pristine and laser-treated ink-coated wood substrates have dramatically different surfaces. The laser passage generates a highly interconnected porous structure, thanks to a combination of factors which include the intumescent behavior of tannic acid^35^, thermal decomposition processes, and the formation of volatile products^36^. Raman measurements of this structure, performed at different positions (Fig. 3e), revealed that the graphitization process was most successful on the top layer. The pronounced G- (~1580 cm^−1^) and 2D-peaks (2680–2690 cm^−1^), together with a small D-peak (~1345 cm^−1^), indicated the presence of turbostratic graphene, with partially graphitized carbon domains localized within the first micrometers. Moving toward the wood substrate, the intensity of both G- and 2D-peaks started to decrease. By contrast the D-peak, which is associated to the presence of defects^32,37^, increased, suggesting a higher structural disorder. The degree of graphitization decreased further until the wood substrate was reached, as indicated by an intense background^38^.

These results were in good agreement with those obtained from wide-angle X-ray diffraction (WAXD), where the rotational and translation disorder of *sp*^2^-hybridized graphene layers suggested this porous microstructure to consist mainly of turbostratic graphene (Supplementary Fig. 5). However, samples treated with high fluence parameters showed emerging (hkl) reflections, indicating partial graphitization^31,36,39,40^. High temperatures during the thermal graphitization of organic materials usually result in both higher degrees of graphitization and bigger crystallites (from 5 to 25 nm)^31^. From the integrated intensity ratio *I*_*D*_/*I*_*G*_ (Eq. (1)), we calculated for our nanographite crystals an in-plane crystallite size *L*_*a*_ = 22 nm, in good agreement with the value estimated from the (002) reflection in the WAXD diffractogram (≈25 nm, Eq. (3)) and with literature reports^11,41,31^. By contrast, using a lower laser fluence decreased the quality of the LIG materials produced (Supplementary Figs. 6–9) resulting in carbon products with a less pronounced graphitic structure (lower stacking order) and more disordered (turbostratic) graphene layers with small in-plane crystallite size *L*_*a*_ ~7 nm (for details, see the Supplementary Information).

A closer structural inspection confirms that the top surface displays the highest degree of graphitization. The morphology of the top surface stands out from the underlying porous carbon (Fig. 3d, g, h). This may be a direct consequence of the different spatio-temporal laser interaction with the surface and bulk of the ink layer. The top surface, being the most exposed to the beam, is expected to absorb the highest amount of energy and consequently to be heated up the most^36^. As shown by energy-dispersive X-ray spectroscopy (EDX), iron is distributed over the entire surface (Fig. 3h). However, its concentration seems to be lower in the areas most exposed to the laser beam (Fig. 3h, Supplementary Table 2). We note here that the volatilization of iron during high-temperature graphitization has been reported before^42^. Observed at higher magnification (Fig. 3d), the porous microstructure appears to be decorated with particles, which transmission electron microscopy (TEM) revealed to be made of a dense iron-rich core surrounded by a lighter carbon foam (Fig. 3i–k)^43,44^. From the WAXD diffractograms, the distribution of iron carbide Fe_3_C (*θ* = 43.9°, 44.6°, 45°) and of iron oxides Fe_x_O_y_ (*θ* = 43.1°)^45,46^ within the turbostratic structure could be inferred, but their univocal identification was prevented by the relatively low peak intensities and a diffraction band overlap at *θ* = 40–50°. The presence of these species is also suggested from X-ray photoelectron spectroscopy (XPS) (Supplementary Fig. 10), more precisely by three signals in the Fe 2*p* area, respectively at 710.8 (Fe^2+^ 2*p*_3/2_), 713.6 (Fe^3+^ 2*p*_3/2_) and 724 eV (Fe^2+^ 2*p*_1/2_) and a peak at 530 eV (Fe-O) in the O 1*s* area^47^. In the C 1*s* plot, a pronounced asymmetric peak at 284.5 eV (*sp*^2^ carbon) and its π-π* satellite indicate graphitic carbon^48^. The shift toward higher eV and the peak broadening within the C 1*s* area is indicative of the presence of iron carbide and oxidized iron species^49,50^, as well as of disordered carbon^48^. The calculated carbon and iron yields for spruce, beech, balsa, oak, and paper samples are summarized in Supplementary Table 2.

Moreover, both the Raman peaks (Supplementary Fig. 6) and intensity ratios (Supplementary Fig. 7) of the top-surface material showed a striking resemblance with materials obtained by treating organic precursors at very high temperatures, around 2500–3000 °C^11,31,51^. This is remarkable, since such temperature values could hardly be reached by our laser system even with a power up to 13 W. We note here that the bright spark arising from the lasing of ink-coated samples (Supplementary Movie 1, Supplementary Fig. 11) is indicative of the localized generation of high temperatures resulting from ink-laser interactions^41^.

Although beneficial for the graphitization process, the localized generation of high temperatures exceeding the decomposition temperature for cellulose and hemicellulose (above 300 °C) could have a negative influence on wood’s mechanical properties^52^. Since converting large-area wood veneers into conductive IC-LIG materials without affecting their mechanical properties is especially important for prospective applications, we performed tensile tests on both native and lased ink-treated spruce and beech veneers. The tensile strength was not reduced as a result of our IC-LIG process (Supplementary Fig. 12).

### Preliminary investigation of IC-LIG mechanism

It is known that certain transition metal cations, such as iron, can have a beneficial effect on the hydrothermal carbonization and pyrolytic graphitization of organic materials, including wood, thanks to thermo-catalytic effects^13,35,51,53–57^. Since our ink contains iron, it is reasonable to hypothesize that thermo-catalytic processes could have promoted efficient laser-induced graphitization already at temperatures between 1200 °C and 1600 °C, well within the expected reach of our lasing parameters^13,51^. For this reason, we call our approach iron-catalyzed laser-induced graphitization (IC-LIG).

To better understand the role of iron in IC-LIG, we investigated wood coated with an iron-free ink i.e., containing only tannic acid, gum arabic and glycerol. Fourier-transform infrared (FTIR) measurements indicated that the light absorption in correspondence of our laser emission  (1060 cm^−1^) of wood coated with our iron-tannic acid ink is much higher compared to that of native wood and of wood coated with the iron-free ink (Supplementary Fig. 13). For wood substrates coated with the iron-free ink, at least two lasing steps were necessary to develop a measurable electrical conductivity (with sheet resistivity values around 60–70 Ω ◻^−1^) with the same laser parameters used to treat iron-tannic acid ink-coated wood. The final product was an irregular carbon foam (Supplementary Fig. 14), devoid of the nano-features observed when using the iron-tannic acid ink. According to the WAXD results, this carbon structure remains completely amorphous even after two laser-engraving steps (Supplementary Fig. 5). Tannic acid is a well-known carbon precursor for high-temperature hydrothermal and pyrolytic carbonization processes^35,58–60^. Our results confirm that this holds for laser-induced graphitization too. They also highlight the crucial role of iron catalysis in promoting its more efficient conversion into high-quality, highly conductive graphite-like materials.

The mechanism for bulk iron-catalyzed thermal graphitization of biomass is already known^13,53,54,57,61–64^ and, at least along its general lines, should also apply to our process. Based on this interpretation, the iron-tannic acid complex would be decomposed under the laser first into amorphous carbon and iron oxide nanoparticles, followed by conversion into iron carbide Fe_3_C by carbothermal reduction. Once the Fe_3_C nanoparticles have reached a critical size, the catalyzed graphitization of amorphous carbon could start. We assume that the graphitization processes and the formation of iron oxide nanoparticles would start already in the heat-affected zone, that is, within the focal plane of the laser, where the temperature can reach  up to 350 °C^20^. However, because our surface laser treatment is extremely fast compared to more conventional bulk graphitization processes, we expect the growth of Fe_3_C particles to be constrained by the time of exposition to high temperatures. Indeed, TEM images show that most of these particles are smaller than 20 nm, and embedded in carbon foam. Thus, the analogy between this mechanism and our process might hold only for the initial moments. Laser-matter interactions most probably play a key role for the iron-catalyzed conversion of amorphous carbon into LIG, and further research is needed to clarify this point. Nevertheless, compared to conventional thermo-catalytic graphitization approaches, our approach requires five times less metal (5.6 wt.% instead of up to 30 wt.%) and only a single step, making additional substrate impregnation with fire retardants, pre-charring of the precursor at temperatures between 300–600 °C and heat-treatments under inert atmosphere unnecessary^13,51,65^.

### Fabrication of IC-LIG-wood electronic devices

#### Strain sensor and flexible electrode

State-of-art structural health monitoring systems are expensive, require dedicated instrumentation, and are difficult to integrate into load-bearing elements without compromising the structural performance of the latter^66^. With the interest for mass timber multi-story buildings on the rise worldwide, it is crucial to provide suitable sensor systems for the assessment of load-bearing wood elements for the improvement of structural design and guarantee serviceability^67^. We show here that, by converting large wood areas into conductive IC-LIG while keeping intact the bulk mechanical properties allows us to reach out for such prospective building-scale applications.

**Fig. 4:**
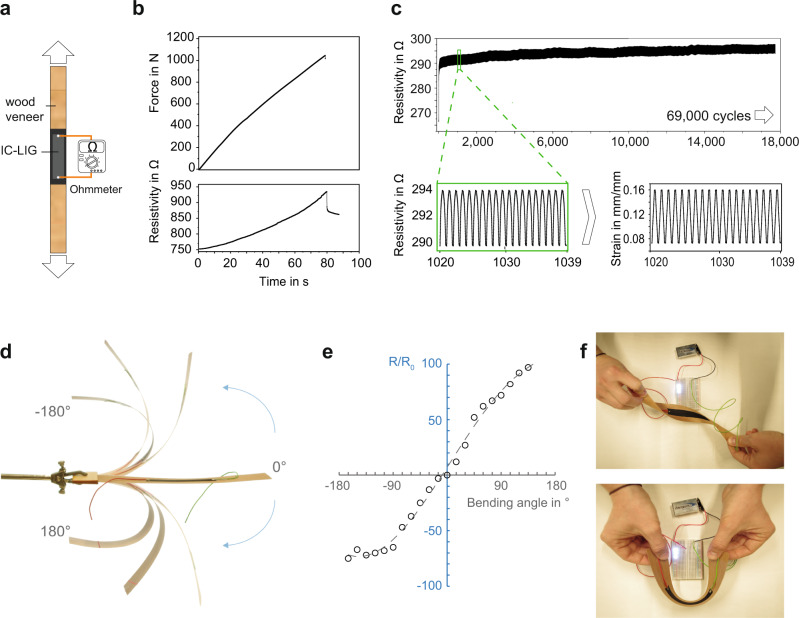
IC-LIG-Wood for strain sensors and flexible electrodes. **a** Schematic representation of a proof-of-concept IC-LIG-wood strain sensor (the arrows indicate the direction of tensile stress application) for measuring the change of resistivity as a function of applied force. **b** Force-time plot with the corresponding resistivity-time plot for a representative IC-LIG-spruce veneer. **c** Resistivity values measured under tensile load cycles with a representative IC-LIG-beech veneer. The measurements were performed for more than 69,000 cycles. The slight increase in resistivity over time is most probably due to the creep of wood veneer during the cycling test. The inset highlights the periodic resistivity change, and the direct correlation between the measured resistivity data from source measure unit with the measured strain values from clip-on extensometer. **d** Picture overlay showing an IC-LIG flexible electrode obtained on a thin cheery wood veneer, which allows for extensive bending angles. **e** Relationship between the bending angle and the associated resistivity change *R/R*_*0*_. **f** Pictures showing the stability of electrical connection even under twisting and bending. Here the flexible IC-LIG-wood electrode is used to connect a battery with a LED light.

To make a proof-of-concept strain sensor device we attached electrodes to the conductive IC-LIG area engraved on spruce and beech veneers. We then measured the change in resistivity during a tensile test under constant humidity conditions (Fig. 4a). As shown in Fig. 4b, the mechanical deformation of the wood veneers resulted in a resistivity increase as a function of strain until failure. Cycling tests confirmed that our LIG structures can sustain >69,000 cycles without significant performance losses (Fig. 4c, Supplementary Movie 4).

Inspired by the durability of our IC-LIG-wood strain sensors, we applied the IC-LIG process on thin (~450 µm) wild cherry (*Prunus avium* L.) wood veneers to produce highly flexible electrodes (Fig. 4d–f). Even after several fast, irregular flexions at high bending angles, their resistivity always returned to the original value (Supplementary Movie 5). The reason for such robustness is the strong connection between the conductive LIG structures with the underlying wood substrate, resulting in a stability unparalleled compared to that achievable with state-of-art carbon-based inks. We demonstrated this point by subjecting both our IC-LIG-wood electrode and a wood veneer coated with a commercial water-based conductive carbon ink to prolonged (up to 30 min) ultrasonication in water. As shown in Supplementary Fig. 15, our IC-LIG electrode kept its electrical performance even under such harsh conditions, while the carbon-based ink completely separated from the wood substrate. Considering the outstanding performance in terms of flexibility and mechanical strength of our IC-LIG electrodes, we envisage prospective applications as flexible sensors and wearable haptic devices for soft robotics and motion tracking.

#### Touch button panel

**Fig. 5:**
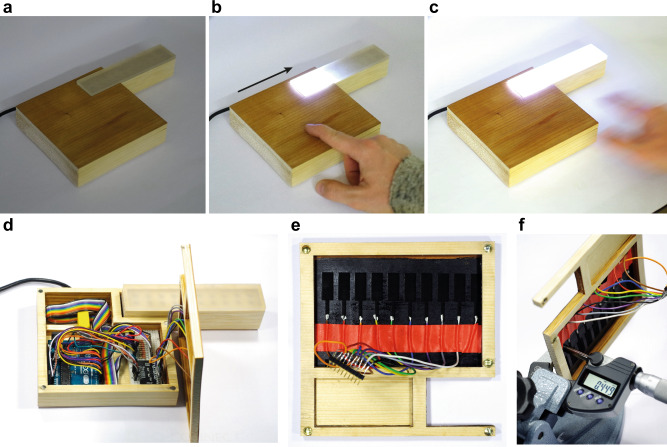
IC-LIG-Wood touch button panel. Capacitive IC-LIG-wood touch button panel made with a thin wild cherry wood veneer. Image sequence showing that the initially “off” (**a**) LED lights can be switched “on” (**b**, **c**) by touching the front native wood veneer surface and moving the finger in the direction indicated by the black arrow. Pictures showing (**d**) the open device, (**e**) the rear wood veneer surface with the conductive IC-LIG touch buttons. **f** Demonstration of how thin the engraved veneer is (maximum thickness 450 µm).

Besides structural and flexible sensors, electrically conductive wooden elements could have useful design applications especially as user interfaces. Large wall panels with controls actuated by capacitive sensing could be used e.g., to switch on and off the lights in a smart home. As a proof-of-concept to demonstrate the potential of IC-LIG for real-world, large-scale wood electronics, we built a touch panel with an array of conductive areas or “buttons” that control a dimmable wooden desk lamp, but the same approach could be applied for large wall panels. Thanks to our IC-LIG approach we can make conductive thin veneers from a variety of wood species (Supplementary Fig. 3), selecting the most adequate for the desired application. Wild cherry (*Prunus avium* L.) wood has a high aesthetic value, hence, we chose it to make a touch panel (Fig. 5) by coating one side of a thin (~450 µm) wild cherry wood veneer with our iron-tannic acid ink and laser-engraving ten areas or “buttons” (Fig. 5e). We connected each laser-engraved touch button to an Arduino microcontroller equipped with a sensor controller (MPR121 breakout board), while each button was considered to a connected LED. The working principle of this kind of device is self-capacitance, in which an electrode forms a capacitor with the ground plane (earth). Here, each laser-engraved conductive area (touch button) is an electrode, and the ground is the wood veneer itself. By applying a voltage to a button, an electric field is generated. When the native surface on the opposite side of a button is touched, the electric field and consequently the capacitance is changed. The sensor controller detects this touch event and switches on or off the associated LED. Thus, the functionalized cherry wood veneer could be used as a decorative touch panel to control a dimmable desk lamp (Fig. 5, Supplementary Movies 2 and 3). Each button could also be assigned to a different function, other than switching lights on and off. Such a sustainable smart veneer, combining the esthetic value and unique haptics of wood, could easily find applications as a user interface in the building sector as well as in the automotive industry (e.g., for car dashboards), but also for point-of-sale applications such as vending machines.

#### Electroluminescent device

**Fig. 6:**
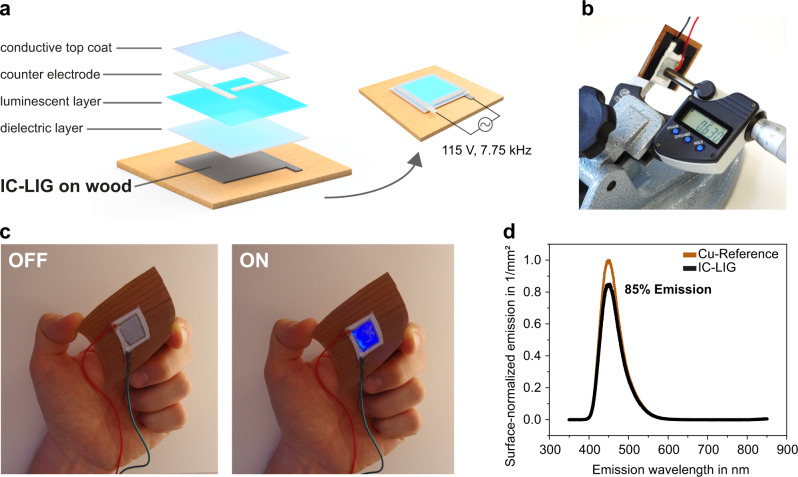
IC-LIG-Wood electroluminescent (EL) device. **a** Exploded view and schematic representation of our IC-LIG-wood EL device. **b** The whole EL device is only 630 µm-thick. **c** Showcasing the operation and flexibility of our IC-LIG-wood EL device. **d** Comparison of light emission efficiency between our EL device, made with an IC-LIG back electrode, and a reference device made with copper-foil as back electrode.

Electroluminescent (EL) flat panel displays are of growing interest for lighting and optical signaling purposes. However, contemporary research efforts that aim at making EL devices more sustainable are limited to the use of biobased materials (e.g., gelatin^68^ and cellulose^69^) as substrates, while the conductive back electrodes remain metal-based (e.g., copper foil, silver paste^68,69^, indium tin oxide ITO^70^). The great environmental benefits of carbon-based electrodes over copper, aluminum and silver electrodes have already been demonstrated for closely-related photovoltaic applications^71^. Here we describe the use of a LIG-based back electrode to fabricate an electroluminescent device, an approach, which has not been reported before. We started by fabricating a 20 × 20 mm^2^ IC-LIG back electrode on a thin (~450 µm) cherry wood veneer. Coating this back electrode first with a standard dielectric paste (barium titanium oxide), then with an electroluminescent phosphor layer (manganese-doped zinc silicate), and eventually with a transparent conductive (PEDOT:PSS) top coating, resulted in a thin (~660 µm) flexible EL device (Fig. 6, Supplementary Movie 6). To show the high performance of our IC-LIG back electrode we assembled a control EL device using a standard copper Cu-foil as the back electrode, *ceteris paribus*, and used it for comparison. The luminescent area was comparably homogeneous in both devices (Supplementary Fig. 16), and we proved by direct measurement that using IC-LIG as back electrode resulted in a light emission efficiency up to 85% compared to that obtained with Cu-foil (Fig. 6d). This is a remarkable achievement, especially taking into account the huge differences between IC-LIG and Cu-foil in terms of electrical conductivity (2500 S m^−1^ *vs* 59‧10^6 ^S m^−1^) and morphology (porous 3D-structure, resulting in the formation of a slightly thicker dielectric layer, *vs* flat homogenous surface). Our device emits light already with an operating voltage of 110 V, corresponding to an electric field of ~1.1 V µm^−1^, and a frequency of 7.75 kHz. For comparison, an electric field >5 V µm^−1^ (with an operating frequency >1 kHz) is required to achieve reasonable brightness in conventional flexible EL devices^72^. Furthermore, we observed that by changing the operating voltage and frequency to 325 V and 50 Hz, respectively, the illuminated area became more uniform, and the emitted color changed from blue to light turquoise (Supplementary Fig. 16).

## Discussion

Conventional approaches toward “green” electronics, involving the substitution of plastic substrates with bio-based (e.g., (nano)cellulose) ones, might not be sufficient to tackle the challenges posed by the increasing demand for sustainable electronic products. Inexpensive, abundant and renewable resources must be used not only as substrates but also as conductors, substituting expensive and polluting metals. In this context, conductive carbon materials derived from biological sources could be desirable environmentally friendly alternatives to silver, copper, and aluminum.

Laser-induced graphitization (LIG) is a promising technique for the fabrication of bio-based conductive carbon materials, but conventional processes have great limitations for demanding substrates such as wood and paper, which require multiple lasing steps in inert atmospheres and the use of hazardous fire retardants, and nevertheless result in high substrate ablation and thermal damage. In this work we addressed and overcame these challenges by applying a new approach, iron-catalyzed laser-induced graphitization (IC-LIG). Using an iron-tannic acid ink and a conventional CO_2_ laser, we managed to engrave large (≥100 cm^2^) highly conductive (up to 2500 S m^−1^) LIG structures on thin (~450 µm) wood veneers (and even on paper) with a single laser step in ambient atmosphere, preserving the substrate mechanical properties and compensating the decreased spatial resolution (due to laser beam defocusing) with an increased processing speed. This favorable fabrication strategy, together with the excellent electrical performance of the obtained products, make our IC-LIG approach especially attractive for prospective industrial-scale applications.

We systematically investigated the interplay between ink, substrate and laser parameters for a variety of wood species, characterizing in detail the properties of the obtained LIG materials with state-of-art techniques. Laser-induced graphitization of iron-tannic acid complexes showed highly beneficial effects on the graphitization of wood and cellulose substrates. Our results pointed out the crucial role of iron promoting the formation of highly conductive LIG, and revealed structure-property relationships for the resulting hierarchically porous graphite-like conductive foam. We mapped large-scale samples with novel eddy-current measurements, demonstrating the homogenous electrical conductivity over the entire sample surface.

To highlight the great potential of IC-LIG for the development of large-scale sustainable wood electronics, we showcased a variety of devices, including a highly durable strain sensor (potentially suitable for structural health monitoring), a flexible electrode, a capacitive touch button panel, and an electroluminescent device. This latter is the first reported example of an electroluminescent device made with LIG as back electrode, which shows a performance comparable to that of a copper-electrode reference. Applications for energy storage devices (e.g., batteries and supercapacitors), requiring porous highly conductive metal-rich structures, are also envisaged for our IC-LIG materials. The high robustness and durability make our IC-LIG materials useful also e.g., in the building and automotive sectors.

## Methods

### Materials

Tannic acid (source: Chinese natural gall nuts), iron(III) citrate (technical), and glycerol (99+%) were purchased from Sigma-Aldrich. Gum arabic (spray dried) was purchased from Spectrum Chemical. Carbon-based aqueous ink (Bare Conductive^®^, UK) and silver paste (EM-Tec, AG44, Micro to Nano, NL) All chemicals were used as received. Sliced veneers were obtained from Norway spruce (*Picea abies*), beech (*Fagus sylvatica*), oak (*Quercus robur* L.), balsa (*Ochroma pyramidale*), wild cherry (*Prunus avium* L.), and ash (*Fraxinus excelsior*). Rotary cut veneers were obtained from birch (*Betula pubescens*) and maple (*Acer pseudoplatanus* L.). All veneers had a thickness of about 1 mm, unless otherwise stated. Deionized water (DI) was thoroughly used unless otherwise stated.

### Preparation of the iron-tannic acid ink

Tannic acid (33 g) was dissolved by adding it in small portions under stirring (500 rpm) to 72 g of deionized water pre-heated to 60 °C. With continued heating and stirring, 5 g of gum arabic, 8 g of glycerol, and eventually 7 g of iron(III) citrate were added sequentially in small portions to ensure their complete dissolution. The resulting iron-tannic acid ink was cooled down to room temperature under stirring and stored at room temperature until use.

### Ink deposition on wood and cellulose paper substrates

The iron-tannic acid ink was applied on thin wood veneers and paper (Whatman) using a commercial paintbrush. Two to three layers of ink were applied to obtain a homogenous coating. The samples were then stored for at least 12 h at 20 °C and 65% RH before use.

### Laser treatment

Samples were treated with a commercial 10.6 µm CO_2_ laser engraver (Speedy 300, Trotec). The laser had a maximum power of 60 W and a maximum scan rate of 3.55 m s^−1^. The used parameter for the laser treatment depended on the substrate. Hence, power ranged between 15 and 30% with scan rates between 150 and 350 mm s^−1^, an image density of 1000 pulses inch^−1^, and a defocus up to 5 mm (resulting beam diameter 0.4 mm). To study the effect of laser fluence on graphitization (Supplementary Figs. 5–9): a laser power of ~13 W with an engraving speed of 200 mm s^−1^ were the parameters used for high fluence (HiF) samples, while for low fluence (LoF) samples a laser power of ~12 W and a scan rate of 270 mm s^−1^ were used.

### Characterization techniques

For electrical properties characterization, squares of size 40 × 40 mm^2^ were laser-engraved on 50 × 50 mm^2^ wood veneer samples. Sheet resistance was measured using a four-point probe (SD-800, NAGY) and electrical resistivity measurements using a source measure unit (2450, Keithley Instruments, US). Sheet resistance maps were obtained using the EddyCus® TF map 2525SR automated sheet resistance mapping device. The samples were mapped with a scanning pitch of 0.25–0.5 mm. The device was calibrated using 50 × 50 mm calibration samples based on NIST reference standards.

Raman spectroscopy was performed with a confocal Raman microscope (Renishaw InVia) using a 532 nm laser, an objective (Zeiss, 20×) and an 1800 l mm^−1^ grating. The integral exposure time was 3 s for 10 accumulations covering a spectral range of 1220–2790 cm^−1^ with 2 MW laser power for single point measurements. Data of single point measurements of the cross section and top view measurements were evaluated using the software OriginPro 2019 (version 9.6.0.172, OriginLab Corporation, US). For comparing cross section spectra, data were normalized (0,1). For analyzing the FWHM of top view measurements, the peak analysis tool in OriginPro software was used.

As mapping parameters, an integration time of 3 s (single spectrum acquisition) with 2 MW laser power and a step width of 500 nm were used in the Streamline HR mode. After data acquisition, a baseline correction and cosmic ray removal filter were applied using the Wire 3.7 software (Renishaw UK). For chemical imaging, data were exported into CytoSpec (v. 2.00.01), a commercially available MatLab-based software. The integrated intensity of the G-peak band (1575–1585 cm^−1^) was used to obtain a color-scaled representation of the Raman map (200 × 200 µm^2^).

Crystallite size L_a_ was calculated^11^ using Eq. (1), in which the intensity ratio between the D- and G-peaks is inversely proportional to crystallite size:

1 $$\frac{{I}_{D}}{{I}_{G}}=\frac{C\,({\lambda }_{L})}{{L}_{a}}$$

The wavelength-dependent pre-factor *C*, for *L*_*a*_ expected to be >2 nm, following the suggestions of Matthews et al. can be expressed as follows (Eq. (2))^73^:

2 $$C\,\left({\lambda }_{L}\right)\approx {C}_{O}+{\lambda }_{L}{C}_{1}$$

where *C*_*O*_ = −12.6 nm and *C*_*1*_ = 0.033, valid for 400 nm < *λ*_*L*_ < 700 nm.

Microstructural measurements were performed using a digital optical microscope (Keyence VHX‐6000, Keyence, JP) and open source image analysis software ImageJ (1.53e). Smooth surfaces of cross sections were prepared using a rotary microtome (Leica RM2255, DE). High-resolution micrographs of laser-treated surfaces and cross sections were taken with the in-lens detector of a field-emission scanning electron microscope (SEM, Leo Gemini 1530, Carl Zeiss AG, DE) driven by an accelerating voltage of 2 kV. Energy-dispersive X-ray spectroscopy (UltraDry II, Thermo Fisher Scientific GmbH, DE) driven by a 20 kV acceleration voltage was used to determine the local concentrations of iron and carbon.

Transmission electron microscopy (TEM) imaging was performed with JEM 1400 (JOEL, JP) with an accelerating voltage of 120 kV. Particles were scraped off the samples, suspended in ethanol, deposited on 400 mesh copper grids and subsequently air-dried.

Wide-angle X-ray diffraction (WAXD) was performed with an X-ray diffractometer (Xpert Pro, Panalytical, UK) equipped with a Soller slit and Cu*-K*$\alpha$_*1*_ radiation source (*λ* = 1.540598 Å) operated at 40 kV and 40 mA. Each scan was done in gonio mode with 2$\theta$ angle ranging from 5° to 70° in 0.016° steps. The analysis of crystallite size *L*_*a*_ was performed for spruce and paper samples by applying the Scherrer equation (Eq. (3)):

3 $${L}_{a}=\frac{1.84\lambda }{{B}_{1/2}\,\left(2\theta \right){{\cos }}\theta }$$

where *B*_*1/2*_ (2θ) (in radian units) is the full width at half maximum of the (101) peak.

The obtained diffraction profiles showed broad peak bands, due to the translational and rotational disorder of the sp^2^-hybridized graphene layers (turbostratic arrangement) resulting in unreliable (hkl) reflections and making them unsuitable for estimating crystallite sizes following the Scherrer equation. Thus, calculating the crystallite sizes with diffraction patterns from samples treated with lower laser fluence was impossible. It is therefore suggested to estimate the crystallite sizes using a fitting approach of scattering profiles developed by Ruland and Smarsly^11,31,36^. However, samples treated with high laser fluence showed more distinct (002) and (101) peaks which allowed a simple estimation using Eq. (3).

X-ray photoelectron spectroscopy (XPS) was performed with a SPECSTM spectrometer (SPECS GmbH, Germany) using a Mg Kα X-ray source (*λ* = 1253.6 eV) with a power of 300 W. The measurements were made at room temperature. Each sample was measured at three spots. The investigated area was typically 10 × 7 mm^2^. Survey spectra were acquired over a binding energy range of 0–1000 eV at pass energy of 30 eV and resolution of 0.5 eV step^−1^. High-resolution spectra of C 1*s*, O 1*s* and Fe 2*p* were an average of three scans acquired at a pass energy of 20 eV and resolution of 0.05 eV step^−1^. The spectra were collected in the same order for each sample (survey, C 1*s*, O 1*s*, Fe 2*p*). The CasaXPS software was used for background subtraction (U 2 Tougaard-type), peak integration, quantitative chemical analysis and deconvolution. The C 1*s* (C–C *sp*^2^/*sp*^3^ overlap) peak at 284.5 eV was used to calibrate the binding energy scale. The C 1*s* area was deconvoluted in four main signals, 284.5 (C–C, C–H), 285.8 (C–O), 287.3 (C=O), and 289.1 eV (O–C=O) assigned to *sp*^2^ and *sp*^3^ carbon, besides a *π*-π* satellite peak (292.7 eV) and two plasmon loss peaks at 290.8 and 295.3 eV. The survey spectra shows a high amount of carbon for all samples, ranging from 73–83 at.%, which is in good agreement with literature values^36^. The amount of iron is relatively low for both wood (between 1.0 and 2.6 at.%) and paper (~3.3–3.5 at.%) samples, in accordance with EDX results (Supplementary Table 2, Fig. 3h, and Supplementary Fig. 17). The differences observed within the absolute values amongst the samples are possibly related to topographic effects^48^.

Fourier-transform infrared spectroscopy (FTIR) was performed in attenuated total reflection (ATR) mode with a FT-IR spectrometer (Tensor 27, Bruker, Switzerland). The spectra were measured with a resolution of 1 cm^−1^ from 4000 to 400 cm^−1^ with 32 scans per measurement.

Mechanical tensile tests were performed according to ISO 527-5 with three sample types (Supplementary Fig. 12a): native wood veneer, ink-coated wood veneer, and laser-treated ink-coated wood veneer. Samples were laser-cut from Norway spruce (*Picea abies*) and beech (*Fagus sylvatica*) thin veneers according to standard sample type A (250 × 15 × 1 mm^3^). A conductive area of 20 × 50 mm^2^ cantered on the sample surface was lased following our IC-LIG approach. End tabs were glued with commercial polyurethane adhesive. Samples were acclimatized to 20 °C and 65% relative humidity for at least one week. Before testing, electrodes (copper wires) were glued with conductive silver paste to the end of the conductive area and connected to a source measure unit (2450, Keithley Instruments, US) for monitoring the resistivity change during tensile test (Supplementary Fig. 12a). Displacement was measured with a clip-on extensometer and used for a comparison with measured resistivity change. All tests were conducted at a climate condition of 20 °C and 65% relative humidity. An initial load of 5 N was applied thereupon samples were tested with a speed of 1 mm min^−1^ until a 50% force drop after the maximal force (*F*_*max*_) had been reached. Cycling test were performed using the same sample measures as we used for tensile tests. Samples were loaded between a nominal strain of 0.5 mm to 1.0 mm with a holding time of 1 s and a speed of 10 mm min^−1^. In parallel, the resistivity change was measured with a source measure unit (2450, Keithley Instruments, US) for monitoring the resistivity change during the cycling test. Due to time constrains, sample was measured for only ~69,000 cycles (69,156 cycles).

### Fabrication of the touch button panel

To make the touch-sensitive veneer for the dimmable desk lamp demonstrator, we engraved areas or “buttons” (Fig. 5e) on a wild cherry (*Prunus avium* L.) thin veneer (ca. 450 µm) using our IC-LIG approach. We glued electrodes (copper wires) with conductive silver paste to the end of each touch button to connect them to a capacitive touch sensor breakout (MPR121, SparkFun) and an Arduino microcontroller (Mega 2560). We attached a panel of ten pairs of LEDs to the Arduino via 220 Ω resistors. Each touch button is connected to a pair of LEDs. The Arduino code is based on Bare Conductive MPR121 Arduino Library^74^ and is available upon reasonable request.

### Fabrication and testing of the electroluminescent device

To make the back electrode, we engraved a 20 × 20 mm^2^ area on ink-coated cherry wood veneer (~450 µm) following our IC-LIG approach. The electroluminescent device was assembled using a commercial kit (LumiLor^®^, Darkside Scientific, USA). Successive layers were applied on the engraved area, as shown in Fig. 6a, starting with a dielectric (barium titanium oxide), then an electroluminescent phosphor (manganese-doped zinc silicate), and finally a transparent top coating of PEDOT:PSS which, together with conductive silver paste, made the counter electrode. We compared the efficiency of our IC-LIG-wood electroluminescent device with that of a reference device, made using copper foil as the back electrode, by measuring the electroluminescence (EL) spectra with an Agilent Cary Eclipse spectrofluorimeter. To ensure accurate measurements all samples were placed in the same position, as close as possible to the detector. The bio/chemiluminescence mode of the spectrofluorimeter was set, collecting spectra from 350 to 850 nm, with a resolution of 0.5 nm and the emission slit set to 2.5.

## Supplementary information

Supplementary Information

Description of Additional Supplementary Files

Supplementary Movie 1

Supplementary Movie 2

Supplementary Movie 3

Supplementary Movie 4

Supplementary Movie 5

Supplementary Movie 6

## Data Availability

The data that support the findings of this study can be found in the article and the Supplementary Information files. Any other relevant data are available from the corresponding author upon request.
