# Supplementary material for: Sustainable wood electronics by iron-catalyzed laser-induced graphitization for large-scale applications
Source: Nat Commun. 2022 Jun 27;13:3680. doi: 10.1038/s41467-022-31283-7 (PMC9237073; doi:10.1038/s41467-022-31283-7)
Supplement: Supplementary file 1 — Supplementary Information [file 41467_2022_31283_MOESM1_ESM.pdf]

Supplementary Information

Sustainable Wood Electronics by Iron-Catalyzed Laser-Induced Graphitization for Large-Scale Applications

Christopher H. Dreimol[1,2], Huizhang Guo[1], Maximilian Ritter[1,2], Tobias Keplinger[1], Yong Ding[1,2], Roman Günther[3,4], Erik Poloni[5†], Ingo Burgert[1,2,], Guido Panzarasa[1,*]*

[1]Wood Materials Science, Institute for Building Materials, ETH Zürich, 8093 Zürich, Switzerland

[2]Cellulose & Wood Materials Laboratory, Empa, 8600 Dübendorf, Switzerland

[3]Laboratory of Adhesives and Polymer Materials, Institute of Materials and Process Engineering, ZHAW Zürich University of Applied Sciences, 8401 Winterthur, Switzerland

[4]Multifunctional Materials, Department of Materials, ETH Zürich, 8093 Zürich, Switzerland.

[5]Complex Materials, Department of Materials, ETH Zürich, 8093 Zürich, Switzerland.

[†]Present address: High Enthalpy Flow Diagnostics Group, Institute of Space Systems, University of Stuttgart, 70569 Stuttgart, Germany.

*Corresponding authors: Ingo Burgert (iburgert@ethz.ch) and Guido Panzarasa (guido.panzarasa@ifb.baug.ethz.ch)

Supplementary Table 1. Data used to make the radar plot in Figure 1c (manuscript).

Parameter	Unit	**This work** [a]	Le et al., 2019	Chyan et al., 2018	Ye et al., 2017
Ablation	µm	**0**	300–900[b]	0–500[b]	0–150[b]
Substrate thickness	µm	**< 200**	1000[b]	250-5000[b]	5000[b]
Conductivity	$S\ m^{-1}$	**2500**	360	150[c]	125[c]
Sheet resistivity	$\Omega\ \square^{-1}$	**20**	10	8	10
Thickness of conductive LIG	µm	**≥ 20**	275	800[b]	800
Laser passes	-	**1**	1[b]	3–5	3–5
Power	W	**max. 13**	0.8	3.75	35–65[c]
Engraving speed	$mm\ s^{-1}$	**350**	10	150[b]	150
Time[d]	min	**2**	70[c]	14.1[c]	14.1[c]
Energy use	W s	**1560**	3360[c]	3172.5[c]	29610[c]
Atmosphere	-	**Ambient**	Ambient	Ambient	N_2/Ar
Inert gas flow	sccm	**no**	-	-	125-175 sccm
Fire retardant	-	**no**	-	Borate, Phosphate	-
Laser source	-	**CO_2**	Femtosecond	CO_2	CO_2
Substrate	-	**Spruce**	Wood	Plywood	Pine

a) Sample was treated with substrate-optimized parameters.
b) Values estimated based on images from main publication and supporting information.
c) Value calculated from information provided in the main publication.
d) Time required for engraving a 20x20 mm^2 square.
e) Not specified.

Supplementary Table 2. Overview of investigated wood and cellulose samples, with their densities and resulting electrical performances as a function of the applied parameter set. For selected samples, the carbon and iron yield was estimated from the XPS atomic percentage values. For comparison, the iron amounts calculated from EDX maps are listed as well.

**Wood species**	**Density[a)] / kg m^{-3}**	**Para-meter set**	**Average sheet resistivity / Ω □$^{-1}$ (st.dev.)**	**XPS Carbon yield [b)] / at.% (st.dev.)**	**XPS Iron yield [c)] / at.% (st.dev.)**	**EDX Iron yield [c)] / wt.% (st.dev.)**
spruce	383.68	LoF	34.56 (2.18)	77.67 (4.83)	1.53 (0.46)	0.59 (0.01) 2.60 ± 0.05
		HiF	24.23 (5.12)			
beech	595.68	LoF	35.51 (3.79)	78.15 (0.63)	2.07 (0.33)	0.56 (0.01) 2.36 ± 0.05
		HiF	28.5 (2.60)			
oak	631.26	LoF	46.81 (2.64)	75.34 (0.59)	1.96 (0.56)	0.59 (0.01) 2.50 ± 0.05
		HiF	30.36 (0.47)			
balsa	225.85	LoF	38.03 (8.43)	77.16 (0.31)	1.66 (0.22)	0.42 (0.01) 1.78 ± 0.04
		HiF [d)]	34.30 (1.04)			
paper	431.81	LoF	61.08 (4.19)	77.35 (0.25)	3.4 (0.08)	nd
		HiF	22.12 (2.22)			
cherry	496.13	LoF	43.40 (5.23)	nd	nd	nd
maple	569.85	LoF	35.23 (3.08)	nd	nd	nd
ash	589.12	LoF	40.37 (3.12)	nd	nd	nd
birch	529.61	LoF	28.93 (1.96)	nd	nd	nd

HiF: High fluence parameters.
LoF: Low fluence parameters.
nd: Not determined.
a) Oven-dried density.
b) Based on XPS atomic percentage values (C 1*s* peak).
c) Based on XPS atomic percentage values (Fe 2*p* peak).
d) High fluence parameters for balsa: 18 W and 300 mm s^{-1}.

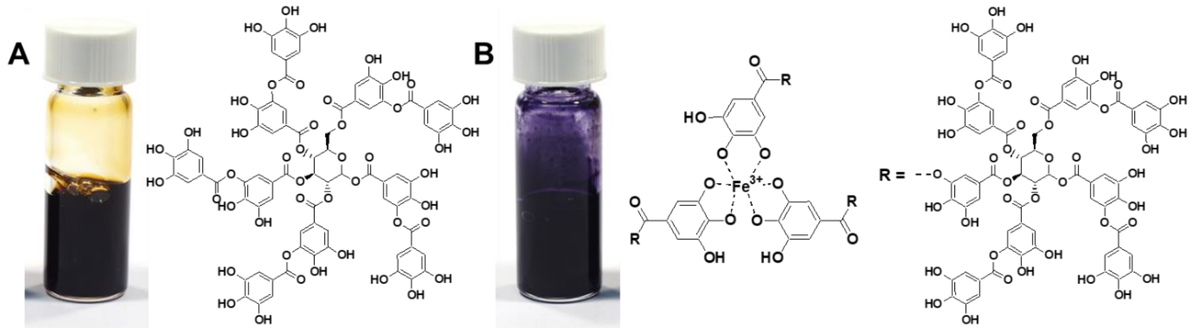

Supplementary Figure 1. Inks used in this study. Photographs of a) the iron-free ink and b) the iron-tannic acid ink, along with the molecular structures of a) tannic acid and b) the iron-tannic acid complex. The deep bluish-purple color of the iron-tannic acid ink suggests a stoichiometry for the complex of two-three gallate ligands per metal ion.[1]

Influence of wood species on IC-LIG performance.

Wood is a challenging material for laser-induced graphitization due to its hierarchical, anisotropic structure and thermal sensitivity. The wood anatomy is characterized at the macroscale by elongated and interconnected tubular cells aligned in the longitudinal direction, while on a micro- and nano scale wood cell walls are made of aligned cellulose fibrils embedded in a matrix of hemicelluloses and lignin. Structural features and differences on the cell and tissue level, *e.g.,* cell type, chemical composition and density variations, determine the most relevant physico-chemical properties of wood, which may vary as much as the diversity of existing wood species.[2] Moreover, the reactivity of each component towards heat can be dramatically different. During combustion, cellulose and hemicelluloses mainly undergo pyrolysis forming volatile products above 300°C,[3] while lignin decomposes at 250–500°C and contributes to char formation.[4-7]

Moreover, the surface of wood is naturally irregular with cut open cells, such as large vessels in oak and ash wood, with up to several tens or hundreds of micrometers in size. Variations on the cell and tissue level together with specific structural features and density variations among wood species (**Supplementary Table 1**) are therefore expected to influence the resulting electrical performance (sheet resistivity as well conductivity). Lasing partially ink-coated wood samples helps to demonstrate the effectiveness of our IC-LIG approach. As shown in **Supplementary Figure 2**, the native side of all tested wood samples had undergone visible damage upon lasing. In the case of native spruce, for example, early wood was completely incinerated while residues of late wood still persisted. Such differences within a single annual ring in a wood sample are expected to result from the associated density variations. Higher density samples from beech and oak wood showed less damage. In contrast, balsa (one of the least dense wood species, *ca.* 225 kg m^{-3}, **Supplementary Table 2** was

completely incinerated by the laser beam. The dependency of wood density on the laser treatment has already been reported for CO_2 laser-cut southern yellow pine, Radiata pine, European redwood and beech,[8] well supporting our findings.

From **Supplementary Figure 2,** it is easy to see how effectively our ink preserves the wood from thermal damages. Even more important is that the same behavior was observed for all the wood species tested, despite the great structural and compositional differences. The ink layer compensates and smoothens surface irregularities as well as the density differences between early- and late wood. All the tested wood species conveniently adsorbed the ink, which was found to penetrate only into the first cell layers. The stability of the ink-wood interface may derive from physical adsorption, mechanical interlocking, and hydrogen bond formation between the iron-tannic acid complex and the hydroxyl groups of hemicelluloses and lignin in the wood cell wall.[9] [10] [11] Thus, the laser treatment resulted in a homogeneous LIGL layer amongst all wood species, irrespective of substrate imperfections, structural differences, laser and grain direction, which can be clearly appreciated from **Supplementary Figures 3-4**.

Another advantage of our IC-LIG approach is that, since the substrate itself (wood or paper) acts as a carbon precursor, the thickness of the ink layer is not as crucial to ensure proper electrical performance. Nevertheless, if the ink layer is too thin the resulting sheet resistivity could be slightly higher, as exemplified by the oak sample whose ink coating was only 20 µm-thick instead of 50 µm as for the other wood species.

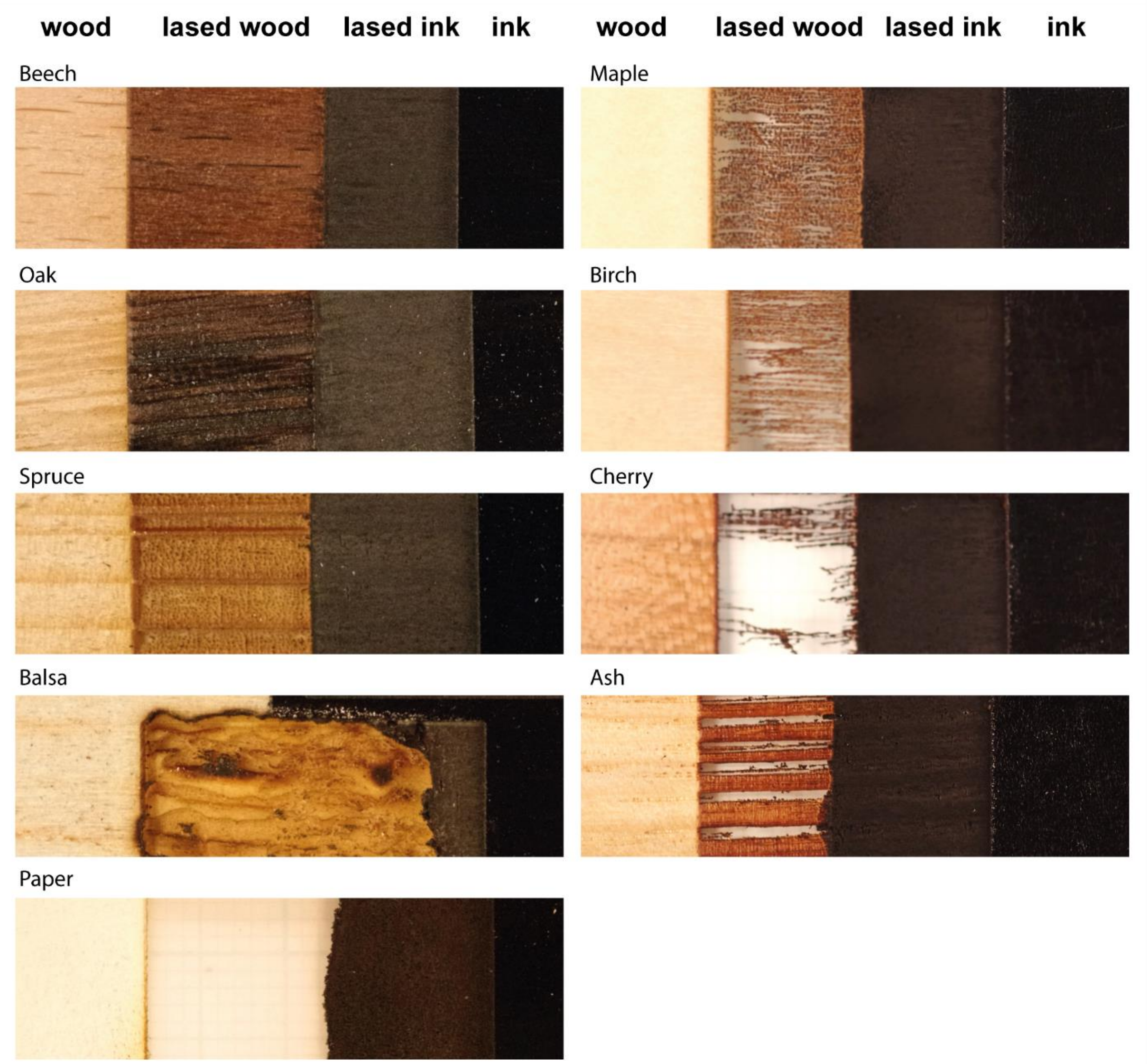

Supplementary Figure 2. Overview of laser-engraved wood samples comparing ink-coated (right) against native surfaces (left). All laser-engraved areas are 40 mm-wide. Laser power 16 W, engraving speed 270 mm s^{-1}, defocus 5 mm, beam diameter 0.4 mm. Uncoated balsa and paper were completely incinerated by the laser. For the other wood species it is possible to see how much early wood was affected compared to late wood. Similar behavior has been reported previously for CO_2 laser-treated southern yellow pine, Radiata pine, and European redwood.[8]

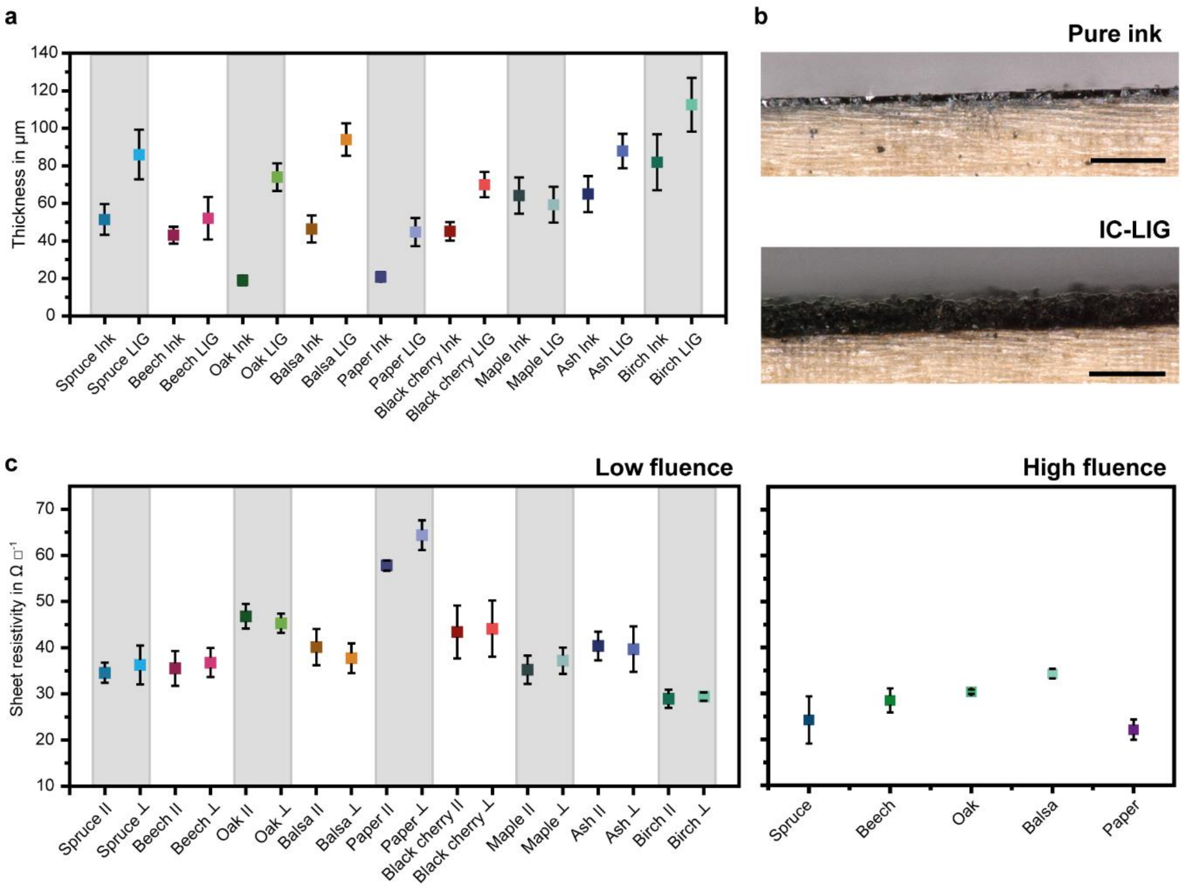

Supplementary Figure 3. Effect of lasing on ink-coated wood. a) Thickness of the ink layer, deposited on different substrates, before ("Ink") and after lasing ("LIG"). Values measured from microscopy images. b) Pictures showing a representative ink-coated wood sample (oak) before ("Pure ink") and after the laser treatment ("IC-LIG"). Scale bar: 200 µm. c) Overview of sheet resistivity (4-point probe) values measured parallel (II) and perpendicular (┴) to grain direction using low fluence parameter set. For some wood species, we applied high fluence parameters that decreased sheet resistivity values. Error bars representing the standard deviation.

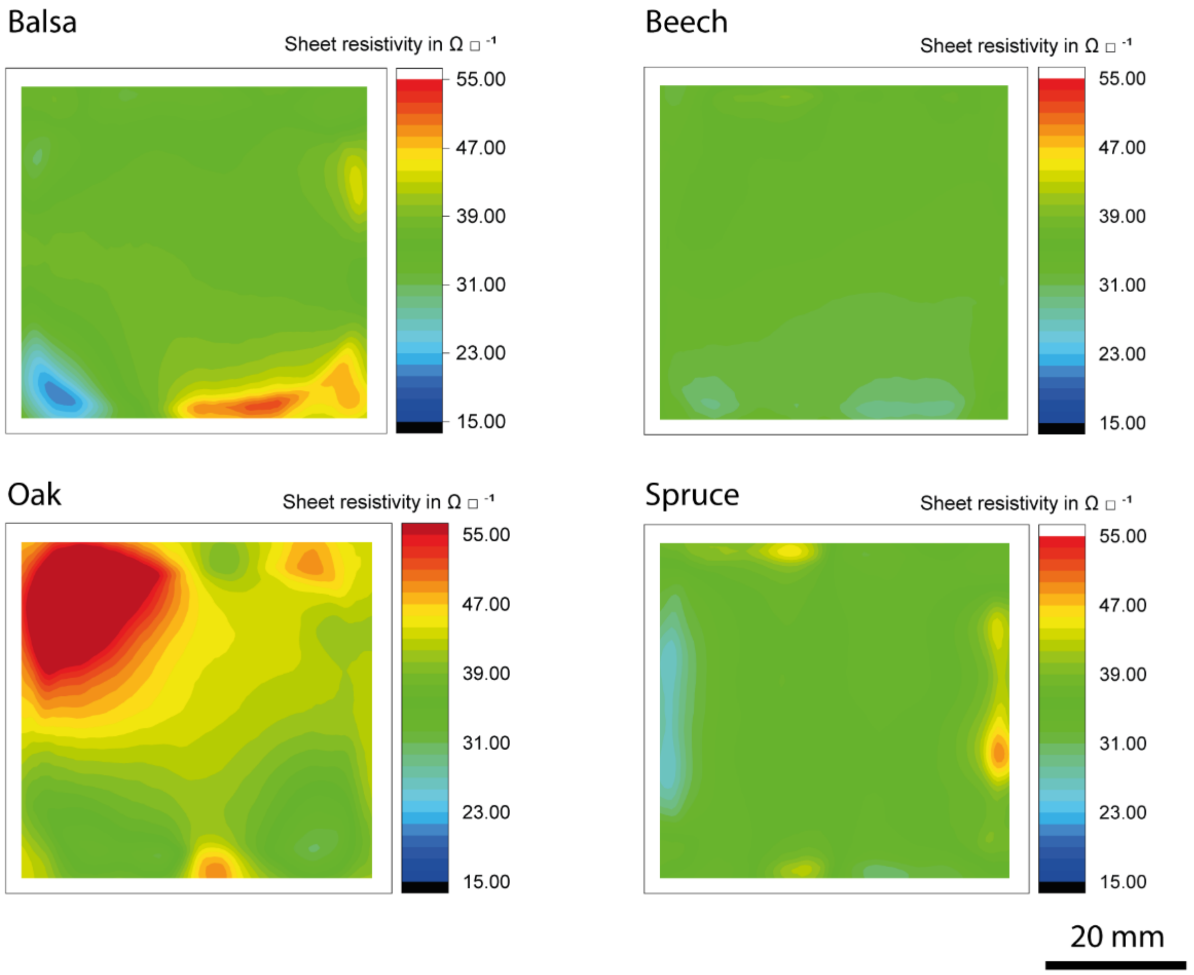

Supplementary Figure 4. Sheet resistivity maps for selected IC-LIG wood samples. The measurements were performed with the eddy current setup, and are in good agreement with those obtained with the 4-point probe.

Influence of laser fluence on graphitization degree.

Improved graphitization using high fluence (HiF) parameters was confirmed by evaluating characteristic Raman features, such as full width at half maximum (FWHM) (**Supplementary Figure 6**), changes in intensity ratios (**Supplementary Figure 7**) and peak position (**Supplementary Figure 8**). In the Raman spectra (**Supplementary Figure 6**), the intensity of the D-peak was reduced and, consequently, the I_D/I_G ratio was lower. Furthermore, the 2D-peak shifted from 2680 to 2696 cm^{-1}, with increased intensity and lower FWHM. The application of high fluence parameters thus resulted in carbon products with higher stacking order as well as more pronounced graphitic structure compared to the more disordered (turbostratic) graphene layers obtained using low fluence parameters (LoF). The in-plane crystalline size L_a (**Equation 1**) was calculated from the integrated intensity ratio I_D/I_G. It has been reported that increasing the temperature during thermal graphitization of organic materials results in both higher degrees of graphitization and bigger crystalline sizes.[12] Using HiF parameters, thanks to the higher energy input, graphitization was not only improved but also resulted in the growth of nanographite crystals, with L_a increasing from ~7 to 22 nm (**Supplementary Figure 9**) in good agreement with literature.[13] [14] [15] We further confirmed these results by means of WAXD. The diffraction patterns of samples lased with substrate-optimized parameters showed emerging (002) and (101) bands (**Supplementary Figure 5**), from which we calculated (**Equation 2**) $L_a \approx 25$ nm (002; reflection mode), in good agreement with the values obtained from Raman measurements.

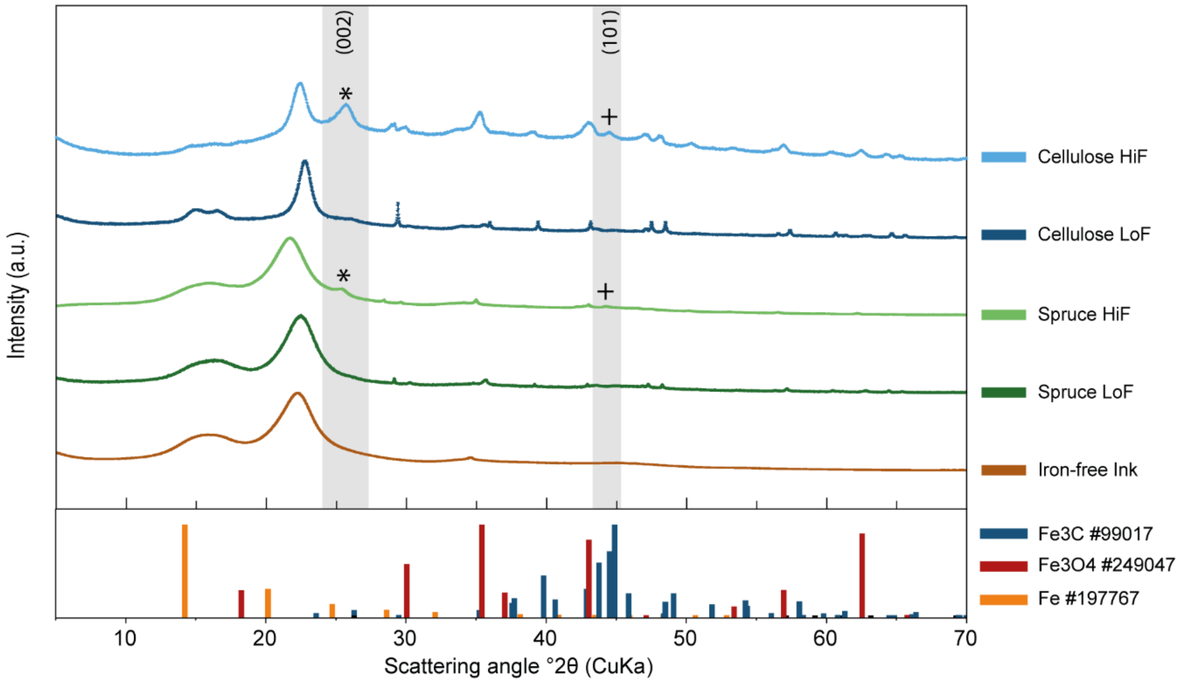

Supplementary Figure 5. WAXD crystallographic analysis of cellulose (reference) and spruce samples, laser-treated with LoF and HiF parameters. Samples treated with HiF parameters showed emerging 002 and 101 peaks (highlighted by the grey boxes), indicated by the star and cross symbols. WAXD data from iron-free shows that the product is a more amorphous carbon structure even after two laser-engraving passages. The main peaks of iron (Fe), iron carbide (Fe_3C), and magnetite (Fe_3O_4) are included in the box below the WAXD data, while the numbers correspond to the associated CCDC crystallographic data. These data can be obtained free of charge from The Cambridge Crystallographic Data Centre via www.ccdc.cam.ac.uk/data_request/cif.

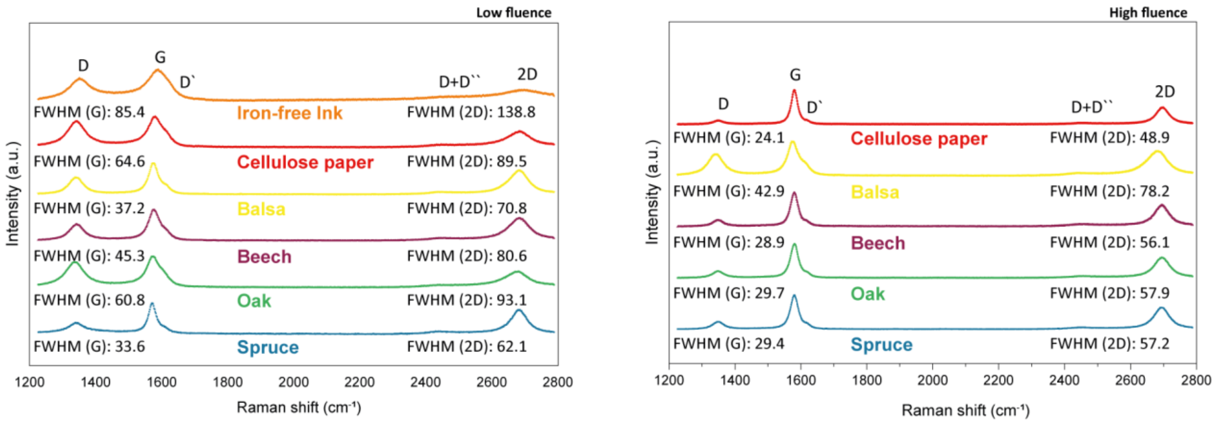

Supplementary Figure 6. Representative Raman spectra of selected wood and paper samples, obtained using LoF and HiF parameters. Improved graphitization by applying HiF parameters led to narrower FWHM for both G- and 2D-peaks and a pronounced intensity decrease of the D-peak.

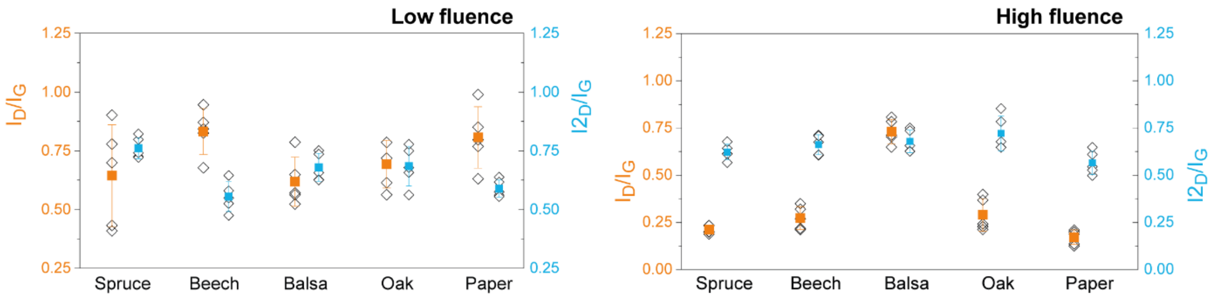

Supplementary Figure 7. Relation between graphitization and the intensity ratio of D, G, and 2D peaks. Comparison between the Raman intensity ratios (I_D/I_G and I_{2D}/I_G) of wood samples lased with LoF and HiF parameters. A decreased I_D/I_G ratio together with an increased I_{2D}/I_G ratio indicate improved graphitization in HiF parameters-treated samples. Filled data points represent mean values with standard deviation, while the blank data points are raw data.

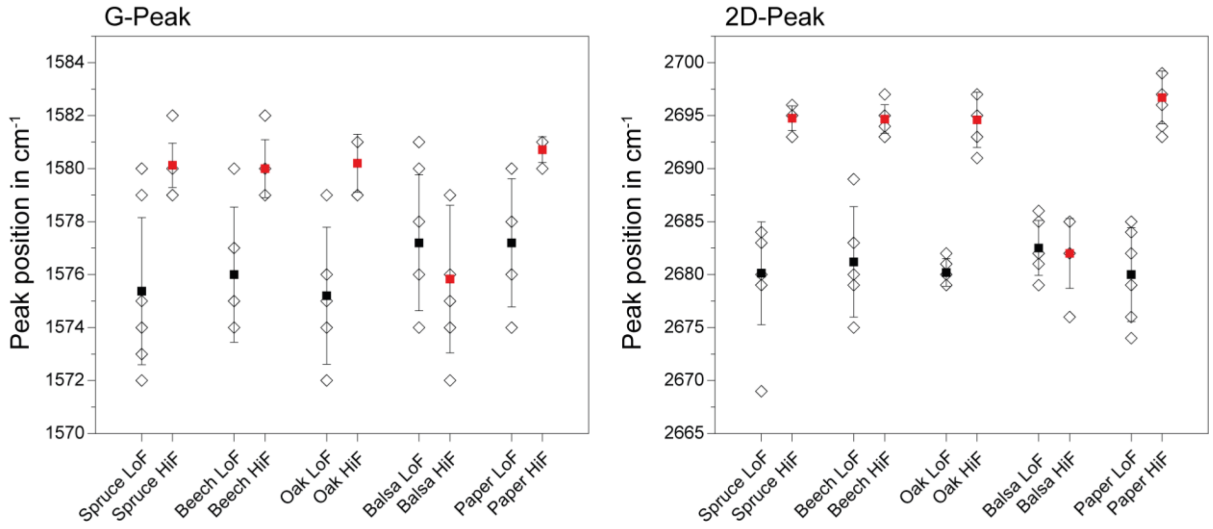

Supplementary Figure 8. Effect of lasing parameters on graphitization. Shifts of Raman G- and 2D-peaks for wood and cellulose paper samples indicate that graphitization increased using HiF (red data points) rather than with LoF (black data points) parameters. The filled data points represent mean values with standard deviation, while the blank data points are raw data.

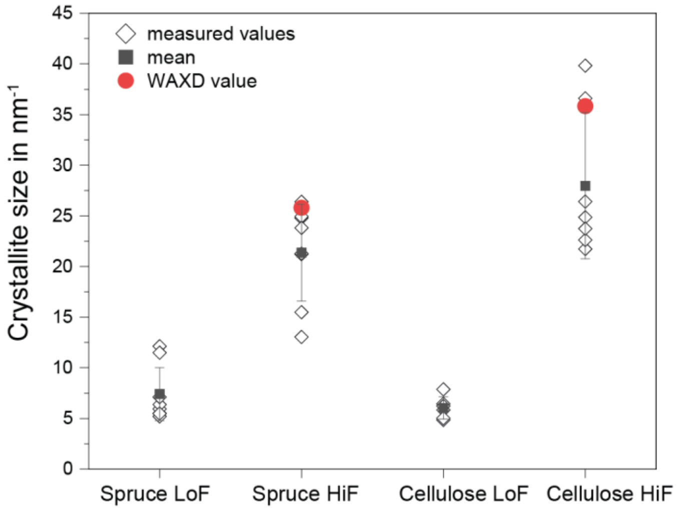

Supplementary Figure 9. Effect of lasing parameters on crystallite size. Crystallite size L_a values estimated from Raman data (**Equation 1**) of spruce and cellulose paper samples treated with LoF and HiF parameters. The higher energy input resulting from the use of HiF led to growth of nanographite crystals, with L_a values in good agreement with those estimated from WAXD data (red data points, **Equation 2**). The filled data points represent the mean values with standard deviation, while the blank data points indicate raw data.

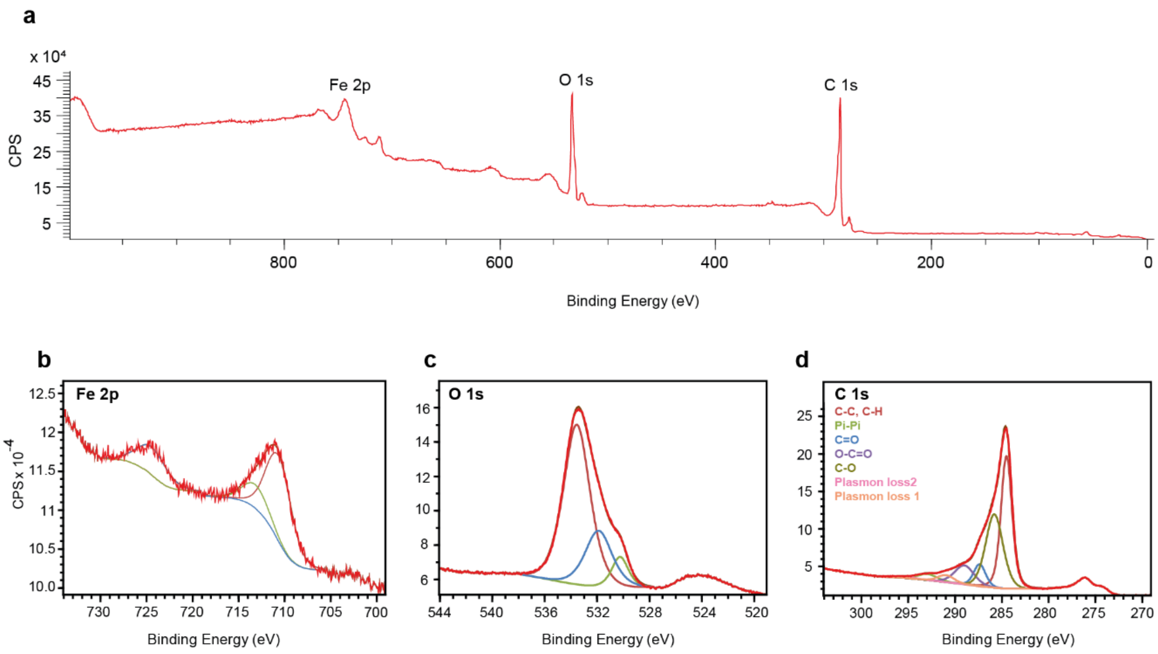

Supplementary Figure 10. X-Ray photoelectron spectroscopy (XPS) of a representative sample of IC-LIG spruce wood. a) Survey spectrum. b-d) High-resolution spectra of b) Fe 2*p*, c) O 1*s*, and d) C 1*s* peaks.

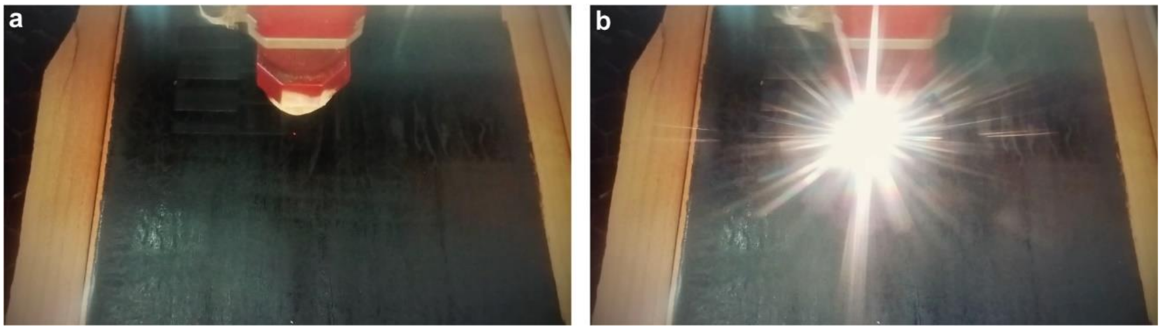

Supplementary Figure 11. Lasing a wood veneer coated with iron-tannic acid ink. a) Laser off, b) laser on. The bright spark arises from the laser-ink interaction, suggesting the generation of laser-triggered thermo-chemical processes.

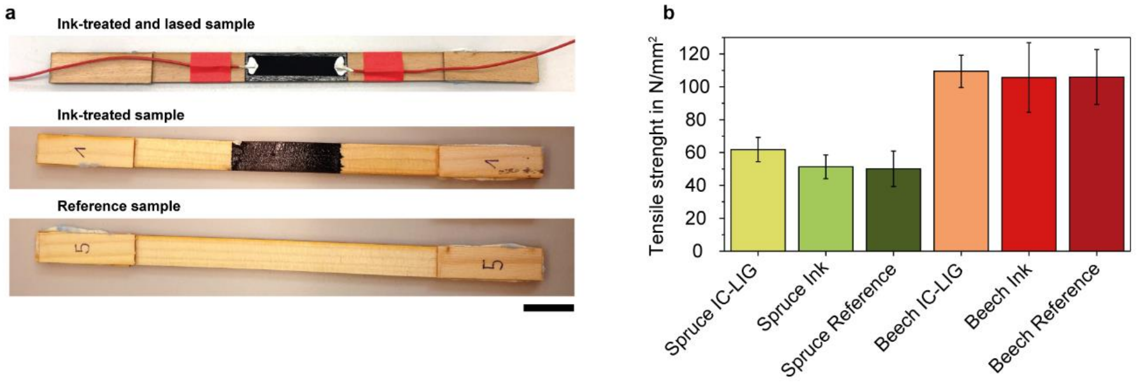

Supplementary Figure 12. Pictures of representative wood samples and tensile strength values. a) Representative spruce samples used for mechanical tests and to measure the change of resistivity as a function of the applied force for tensile and cycling tests. Scale bar: 25 mm. b) Tensile strength of lased samples ("IC-LIG") and ink-treated ("Ink") spruce and beech samples compared with that of reference (native) wood samples. Error bars representing the standard deviation.

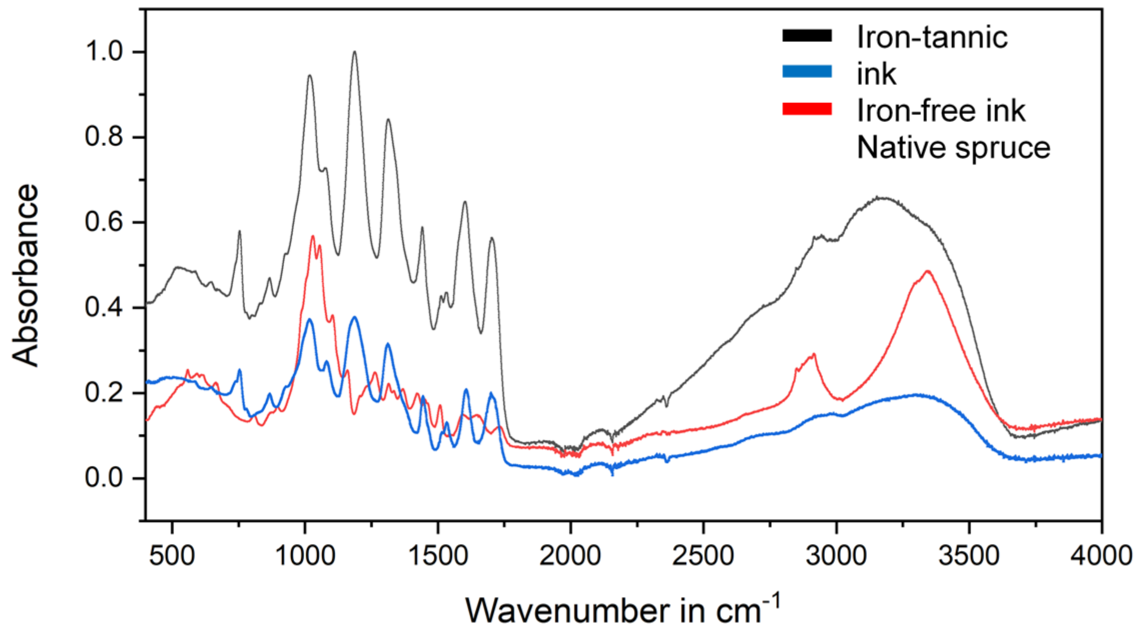

Supplementary Figure 13. Comparison of the FTIR spectra of iron-tannic acid ink-coated spruce (black line), iron-free ink-coated spruce (blue line) and native spruce (red line). Coating the spruce surface with the iron-tannic acid ink results in an increase of absorbance especially in the range of 900-1300 cm^{-1}, in correspondence to our laser wavelength (1060 cm^{-1}).

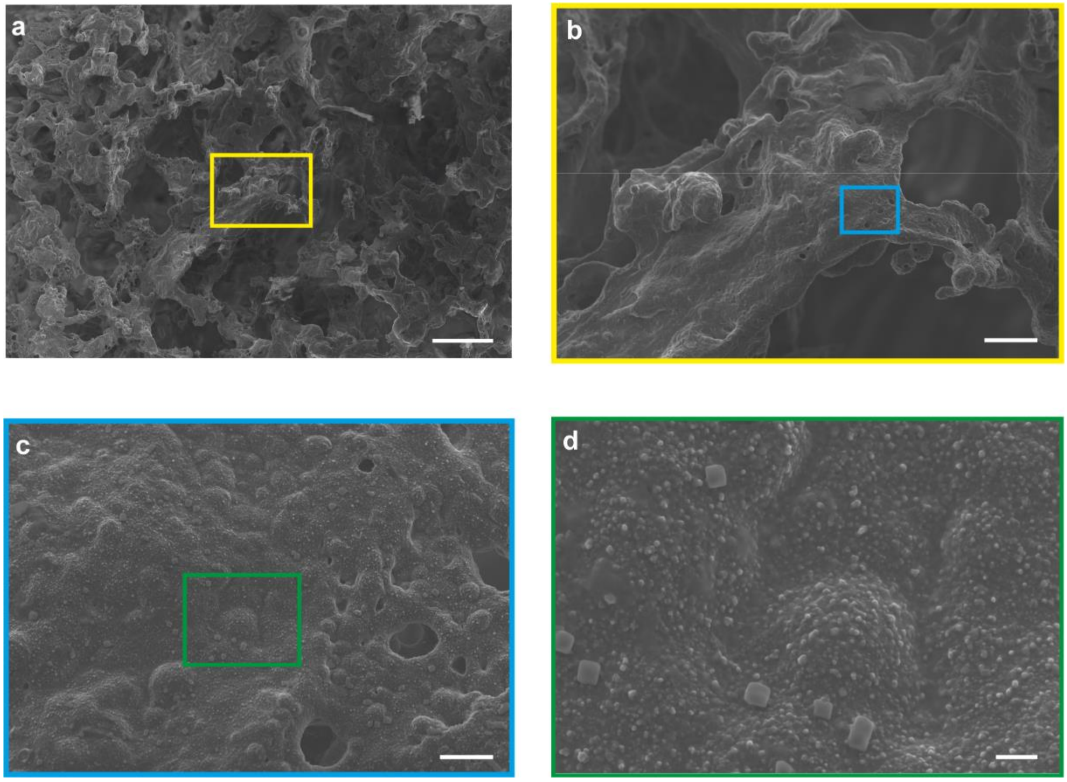

Supplementary Figure 14. SEM images of laser-treated iron-free tannic acid ink on spruce showing an irregular amorphous carbon foam. The surface of laser-treated iron-free ink samples is relatively smooth and does not show significant nanostructuration at higher magnifications, in contrast with what observed for laser-treated iron-containing ink (manuscript, **Figure 3**). Scale bars: a) 100 µm, b) 20 µm, c) 2.5 µm, d) 500 nm.

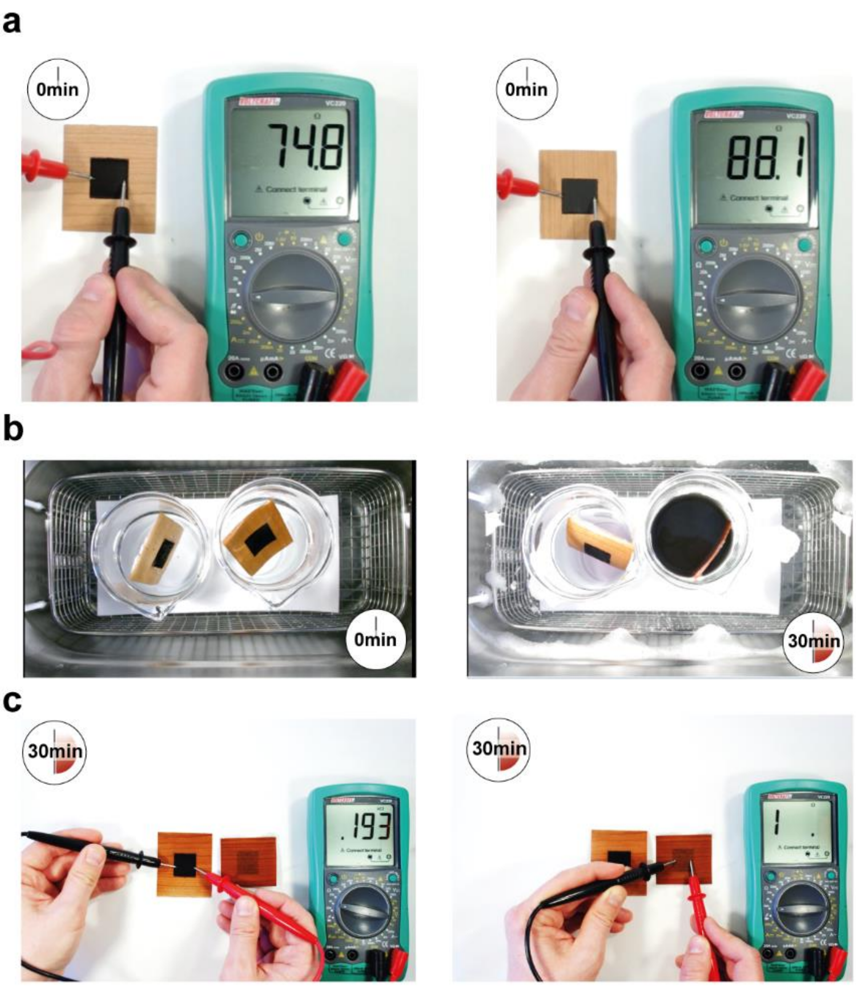

Supplementary Figure 15. Demonstrating the superior robustness of our IC-LIG-wood compared to that of wood coated with a commercial conductive ink. a) Resistivity measurement on an IC-LIG wood sample (left, 75 Ω) and a wood sample coated with a commercial carbon-based ink (right, 88 Ω). Both samples are 20x20mm^2. b) The samples are immersed in water and ultrasonicated for 30 min. While the IC-LIG sample did not show any visible change, the carbon-based ink was detached almost entirely from the wood surface during ultrasonication. c) Resistivity measurement on the samples directly after ultrasonication, showing that only the IC-LIG sample is still conductive (193 Ω, wet state).

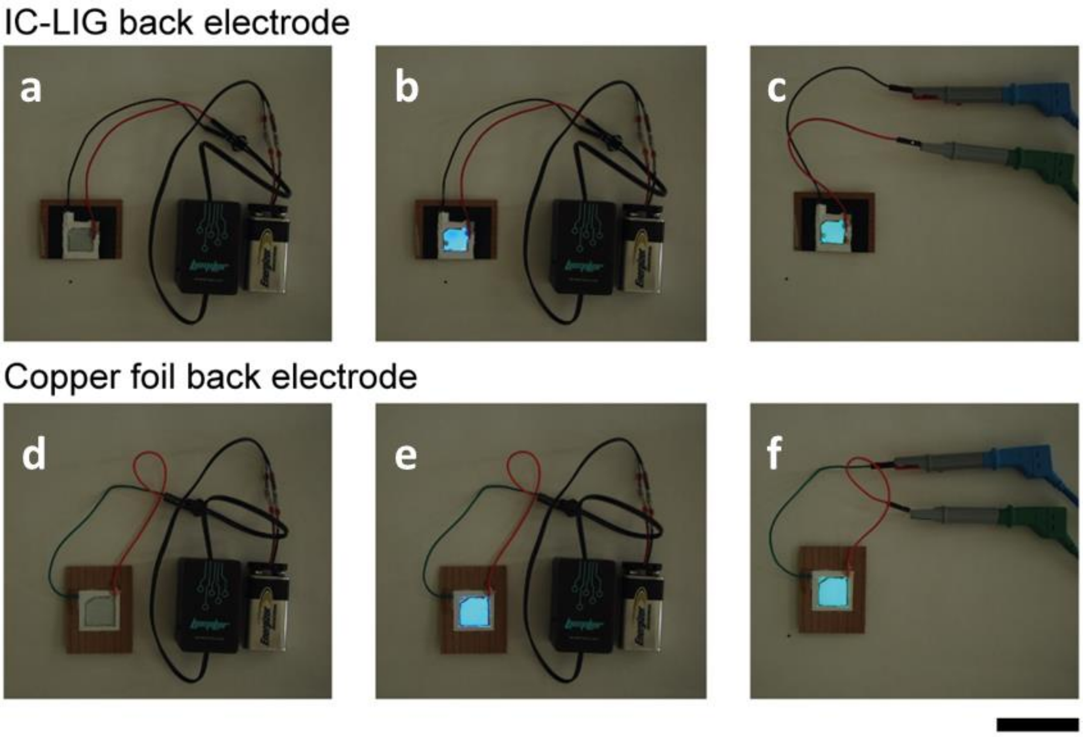

Supplementary Figure 16. IC-LIG-Wood electroluminescent device. Comparison of (a-c) our electroluminescent (EL) device made with an IC-LIG back electrode and (d-f) a reference EL device made with copper-foil as back electrode. b,e) Blue light is emitted from the EL devices with an operating voltage and frequency of 110 V and 7.75 kHz, respectively. c,e) With higher operating voltage and frequency (325 V, 50 Hz), the illuminated area became more uniform and the emitted color changed from blue to light turquoise. Scale bar: 5 cm.

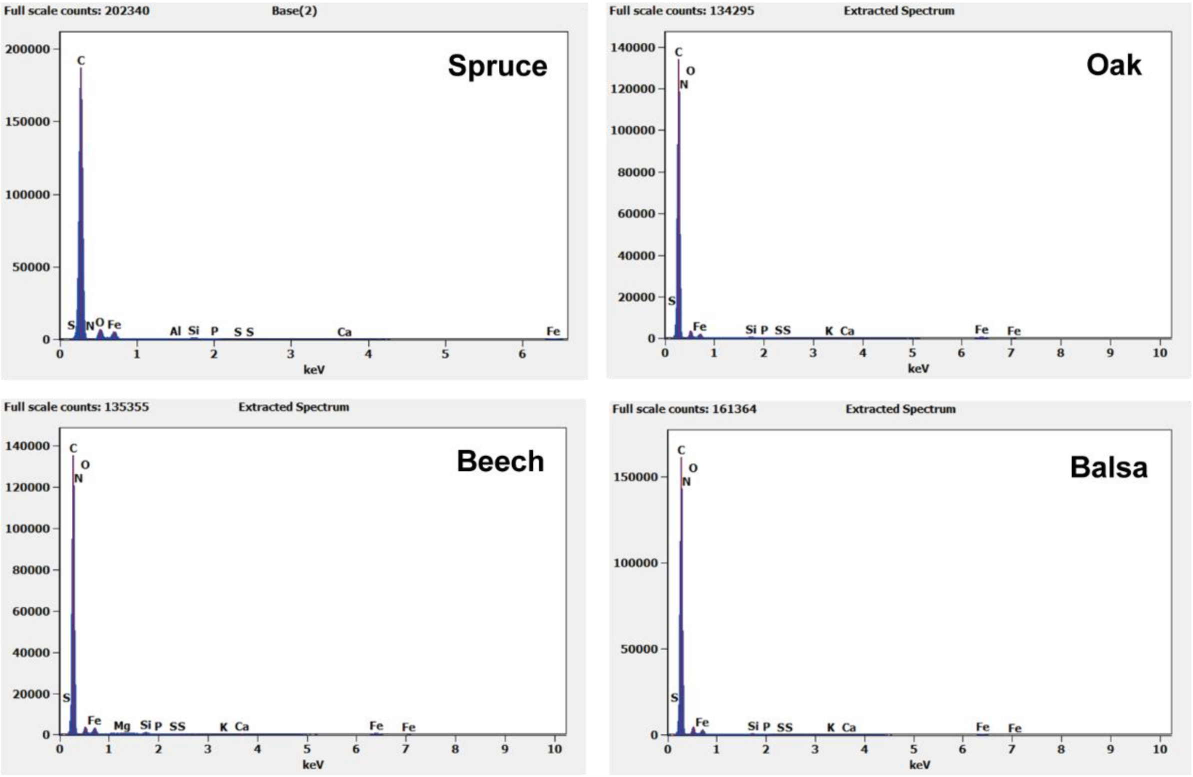

Supplementary Figure 17. EDX spectra of IC-LIG structures obtained on ink-coated wood samples.

References

1 Perron, N. R. & Brumaghim, J. L. A review of the antioxidant mechanisms of polyphenol compounds related to iron binding. *Cell Biochem Biophys* **53**, 75-100, doi:10.1007/s12013-009-9043-x (2009).

2 Byrne, C. E. & Nagle, D. C. Carbonization of wood for advanced materials applications. *Carbon* **35**, 7 (1997).

3 Le, T. S. D., Park, S., An, J., Lee, P. S. & Kim, Y. J. Ultrafast Laser Pulses Enable One - Step Graphene Patterning on Woods and Leaves for Green Electronics. *Advanced Functional Materials* **29**, doi:10.1002/adfm.201902771 (2019).

4 Ye, R. *et al.* Laser-Induced Graphene Formation on Wood. *Adv Mater* **29**, doi:10.1002/adma.201702211 (2017).

5 Chyan, Y. *et al.* Laser-Induced Graphene by Multiple Lasing: Toward Electronics on Cloth, Paper, and Food. *ACS Nano* **12**, 2176-2183, doi:10.1021/acsnano.7b08539 (2018).

6 Guo, H. *et al.* Struvite Mineralized Wood as Sustainable Building Material: Mechanical and Combustion Behavior. *ACS Sustainable Chemistry & Engineering* **8**, 10402-10412, doi:10.1021/acssuschemeng.0c01769 (2020).

7 Poletto, M., Zattera, A. J., Forte, M. M. & Santana, R. M. Thermal decomposition of wood: influence of wood components and cellulose crystallite size. *Bioresour Technol* **109**, 148-153, doi:10.1016/j.biortech.2011.11.122 (2012).

8 Nath, S. *et al.* CO2 laser interactions with wood tissues during single pulse laser-incision. *Optics & Laser Technology* **126**, doi:10.1016/j.optlastec.2020.106069 (2020).

9 Cabane, E., Keplinger, T., Merk, V., Hass, P. & Burgert, I. Renewable and functional wood materials by grafting polymerization within cell walls. *ChemSusChem* **7**, 1020-1025, doi:10.1002/cssc.201301107 (2014).

10 Keplinger, T. *et al.* A versatile strategy for grafting polymers to wood cell walls. *Acta Biomater* **11**, 256-263, doi:10.1016/j.actbio.2014.09.016 (2015).

11 Canevari, C. *et al.* Chemical characterization of wood samples colored with iron inks: insights into the ancient techniques of wood coloring. *Wood Science and Technology* **50**, 1057-1070, doi:10.1007/s00226-016-0832-2 (2016).

12 Sergiienko, R., Shibata, E., Kim, S., Kinota, T. & Nakamura, T. Nanographite structures formed during annealing of disordered carbon containing finely-dispersed carbon nanocapsules with iron carbide cores. *Carbon* **47**, 1056-1065, doi:10.1016/j.carbon.2008.12.029 (2009).

13 Zickler, G. A., Smarsly, B., Gierlinger, N., Peterlik, H. & Paris, O. A reconsideration of the relationship between the crystallite size La of carbons determined by X-ray diffraction and Raman spectroscopy. *Carbon* **44**, 3239-3246, doi:10.1016/j.carbon.2006.06.029 (2006).

14 Lin, J. *et al.* Laser-induced porous graphene films from commercial polymers. *Nat Commun* **5**, 5714, doi:10.1038/ncomms6714 (2014).

15 Schuepfer, D. B. *et al.* Assessing the structural properties of graphitic and non-graphitic carbons by Raman spectroscopy. *Carbon* **161**, 359-372, doi:10.1016/j.carbon.2019.12.094 (2020).
