# Supplementary material for: Sustainable wood electronics by iron-catalyzed laser-induced graphitization for large-scale applications
Source: Nat Commun. 2022 Jun 27;13:3680. doi: 10.1038/s41467-022-31283-7 (PMC9237073; doi:10.1038/s41467-022-31283-7)
Supplement: Supplementary file 2 — Description of Additional Supplementary Files [file 41467_2022_31283_MOESM2_ESM.pdf]

Description of Additional Supplementary Files

File Name: Supplementary Movie 1
Description: Laser treatment of ink-coated cherry wood veneer. The bright spark observed during lasing indicates the localized generation of high temperatures, a promising sign for successful conversion of the substrate into graphite-like materials.

File Name: Supplementary Movie 2
Description: Capacitive touch button panel demonstrator (dimmable desk lamp).

File Name: Supplementary Movie 3
Description: Demonstration that self-capacitance is the mechanism responsible for the functioning of the touch button panel: only by touching the IC-LIG wood veneer, and not the connecting cable, it is possible to switch on/off the LED light.

File Name: Supplementary Movie 4
Description: Cycling mechanical test of an IC-LIG beech wood strain sensor. Even after 15,000 cycles, and after fast and irregular flexions at high bending angles, the resistivity always returned to its ground state demonstrating the IC-LIG durability. Afterwards, the cycling test was resumed. Even after >69,000 cycles, the sample was still intact and working properly.

File Name: Supplementary Movie 5
Description: Demonstration of flexible electrode on thin cherry wood veneer used to connect a battery with a LED light.

File Name: Supplementary Movie 6
Description: First demonstration of an IC-LIG-wood electroluminescent device (operating conditions: ~325 V, 50 Hz).
